# Abnormal ClC-3/TMEM9-mediated endosomal ion transport in *CLCN3*-associated neurodevelopmental disease

**DOI:** 10.1038/s44321-026-00486-6

**Published:** 2026-07-22

**Authors:** Maya M Polovitskaya, Tinatin Tkemaladze, Lotte Jensen, Sumanta Kar, Rosa Planells-Cases, Emanuele Agolini, Pankaj B Agrawal, Paolo Alfieri, Tahsin Stefan Barakat, Miriam Bertrand, Pamela Bowman, Alice S Brooks, Ange-Line Bruel, Sara Cabet, Karolina Chwialkowska, Giovanna Stefania Colafati, Julian Delanne, Laurence Faivre, Cristina Felton, Ute Grasshoff, Anne-Marie Guerrot, Tobias B Haack, Claudia Haase, Ashley R Helseth, Katrien Janssens, Amjad Khan, Margarete Koch-Hogrebe, Joshua Mandelberg, Elisabeth Mangold, Elene Melikidze, Marije Meuwissen, Ieva Mičule, Brittany Noble, Konrad Platzer, Dmitrijs Rots, Catherin Sarret, Ariane Schmetz, Amelle Shillington, Frédéric Tran-Mau-Them, Dagmar Wieczorek, Gaetan Lesca, Allan Bayat, Thomas J Jentsch

**Affiliations:** 1 Leibniz Forschungsinstitut für Molekulare Pharmakologie (FMP), Berlin, Germany; 2 Department of Molecular and Medical Genetics, Tbilisi State Medical University, Tbilisi, Georgia; 3 Department of Pediatrics, Givi Zhvania Pediatric Academic Clinic, Tbilisi State Medical University, Tbilisi, Georgia; 4 Department of Pediatrics, Danish Epilepsy Center, Dianalund, Denmark; 5 Laboratory of Medical Genetics, Translational Cytogenomics Research Unit, Bambino Gesù Children Hospital, IRCCS, Rome, Italy; 6 Division of Neonatology, Department of Pediatrics, University of Miami Miller School of Medicine and Holtz Children’s Hospital, Jackson Health System, Miami, FL USA; 7 Child and Adolescent Neuropsychiatry Unit, Bambino Gesù Children’s Hospital, IRCCS, Rome, Italy; 8 Department of Clinical Genetics, Erasmus MC University Medical Center, Rotterdam, The Netherlands; 9 Institute of Medical Genetics and Applied Genomics, University of Tübingen, Tübingen, Germany; 10 Clinical Genetics Department, RDUH NHS Foundation Trust, Exeter, UK; 11 Université Bourgogne Europe, CHU Dijon Bourgogne, Laboratoire de Génomique Médicale, Centre Neomics, FHU-TRANSLAD, Centre de recherche Translationnelle en Médecine moléculaire – Inserm UMR1231 équipe GAD, Dijon, France; 12 Pediatric and Fetal Imaging Department, Hôpital Femme-Mère-Enfant, Hospices Civils de Lyon, Lyon, France; 13 Laboratory of Computational Molecular Medicine, Clinical Research Centre, Medical University of Bialystok, Bialystok, Poland; 14 IMAGENE.ME SA, Bialystok, Poland; 15 Oncological Neuroradiology and Advanced Diagnostics Unit, Bambino Gesù Children’s Hospital, IRCCS, Rome, Italy; 16 Université Bourgogne Europe, CHU Dijon Bourgogne, Inserm, CTM UMR1231, équipe GAD, FHU TRANSLAD, Centre de génétique, Centre de référence Anomalies du Développement et Syndromes Malformatifs, Centre de référence Déficiences Intellectuelles de Causes Rares, et Centre de référence GénoPsy, Dijon, France; 17 UF Health Jacksonville Genetics and Genomics, Jacksonville, FL USA; 18 Department of Genetics and Reference Center for Developmental Disorders, Normandie Université, UNIROUEN, Inserm U1245 and Rouen University Hospital, Rouen, France; 19 Helios Hospital Erfurt, Department of Pediatrics, Erfurt, Germany; 20 Atrium Health Levine Children’s Neurology, Charlotte, NC USA; 21 Center of Medical Genetics, Antwerp University Hospital/ University of Antwerp, Edegem, Belgium; 22 Institute of Biological Sciences, Department of Zoology, University of Lakki Marwat Khyber Pakhtunkhwa, Lakki Marwat, Pakistan; 23 Vestische Kinder- und Jugendklinik, Datteln, Germany; 24 Developmental-Behavioral Pediatrics, Private Practice, Los Angeles, CA USA; 25 Institute of Human Genetics, University of Bonn, Medical Faculty and University Hospital Bonn, Bonn, Germany; 26 Children’s Clinical University Hospital, Riga, Latvia; 27 GeneDx, LLC, Gaithersburg, MD USA; 28 Institute of Human Genetics, University of Leipzig Medical Center, Leipzig, Germany; 29 Rīga Stradiņš University, Riga, Latvia; 30 Department of Genetics, CHU de Clermont-Ferrand, Clermont-Ferrand, France; 31 Zotz/Klimas, MVZ für Interdisziplinäre Medizin IDM, Aachen, Germany; 32 Department of Human Genetics, Cincinnati Children’s Hospital Medical Center, Cincinnati, OH USA; 33 Department of Pediatrics, University of Cincinnati College of Medicine, Cincinnati, OH USA; 34 Université Bourgogne Europe, CHU Dijon Bourgogne, Laboratoire de Génomique Médicale, Centre Neomics, FHU TRANSLAD, Centre de recherche Translationnelle en Médecine moléculaire – Inserm UMR1231, équipe GAD, Dijon, France; 35 Heinrich-Heine-University Düsseldorf, Institute of Human Genetics, Medical Faculty and University Hospital Düsseldorf, Düsseldorf, Germany; 36 University Hospitals of Lyon (HCL), Department of Genetics, Member of the ERN EpiCARE, Lyon, France; 37 Pathophysiology and Genetics of Neuron and Muscle, CNRS UMR 5261-INSERM U1315, Université de Lyon-Université Claude Bernard Lyon 1, Lyon, France; 38 Department for Regional Health Research, University of Southern Denmark, Odense, Denmark; 39 NeuroCure Cluster of Excellence, Charité Universitätsmedizin, Berlin, Germany; 40 Present Address: Department of Biochemistry, Universität Zürich, Zurich, Switzerland

**Keywords:** Genetics, Gene Therapy & Genetic Disease, Neuroscience

## Abstract

Endolysosomal abnormalities are particularly detrimental to the nervous system and have been implicated in neuropsychiatric disorders. Key regulators of the lysosomal and endosomal luminal ion homeostasis are CLC chloride/proton exchangers. We report 15 individuals carrying variants in *CLCN3*, encoding a ubiquitous endosomal 2Cl^−^/H^+^ exchanger, and provide updated clinical information for 5 previously reported individuals. Subjects displayed a broad spectrum of neuropsychiatric symptoms, including developmental delay, intellectual disability, and epilepsy. To reveal the pathogenic mechanism, we investigated ClC-3 variants-mediated ion transport and its regulation by the recently discovered inhibitory beta subunit TMEM9. 12/20 missense variants exhibited altered properties and fell into two classes: those affecting the region binding inhibitory TMEM9 carboxy-termini, and those that broaden the voltage range over which ClC-3 conducts ions. Surprisingly, the latter variants also attenuated TMEM9-mediated inhibition. Both classes produced a toxic gain-of-function, as evident from endolysosomal vacuolization by mutant ClC-3/TMEM9 overexpression. Our results expand the genetic and clinical spectrum of *CLCN3-*related disease, provide a solid basis for genetic counseling, and uncover an unexpected link between gating-associated conformational changes and inhibition by TMEM9.

## Synopsis

Loss- and gain-of-function variants of the endosomal chloride/proton exchanger ClC-3 are associated with neurodevelopmental disorders. Identification and characterization of novel variants expands the clinical spectrum of *CLCN3* disease and provides detailed insights into pathogenic mechanisms.

- Most heterozygous missense variants result in a gain of function when studied in co-expression with TMEM9 β-subunits.
- Several variants affect ClC-3 residues close to the binding pocket of the TMEM9 carboxy-terminus that directly blocks the chloride pathway, thereby weakening the block.
- Several other variants, located far from the binding site, affect voltage-dependent gating when studied without TMEM9, thereby enhancing currents at endosomal voltages.
- These variants also weaken TMEM9-mediated inhibition, revealing a link between gating-associated conformational changes and TMEM9 binding.

## Abstract (web summary)

Loss- and gain-of-function variants of the endosomal chloride/proton exchanger ClC-3 are associated with neurodevelopmental disorders. Identification and characterization of novel variants expands the clinical spectrum of *CLCN3* disease and provides detailed insights into pathogenic mechanisms.

## Graphical abstract

**Figure:**
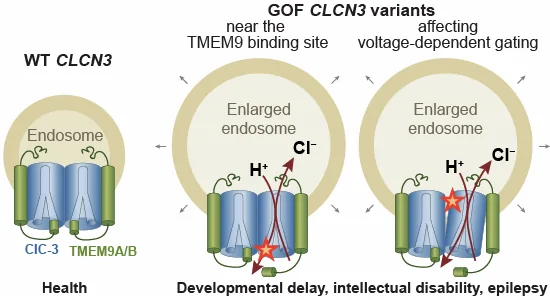

**Figure:**
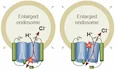

The paper explained **Problem**

Luminal ion homeostasis is essential for endosome and lysosome function, including protein sorting, metabolism, and signaling. Accordingly, mutations in endolysosomal ion channels and transporters cause various inherited diseases, often with prominent neurological manifestations because neurons depend on precise intracellular trafficking and cannot dilute toxic material through cell division.

Five chloride/proton exchangers of the *CLCN* family regulate pH and chloride concentrations in overlapping endolysosomal compartments, and mutations in all of them are linked to disease. Loss of the late-endosomal ClC-3 transporter (encoded by *CLCN3*) causes severe neurodegeneration, while several human variants have been associated with neurodevelopmental and psychiatric disorders. However, the underlying disease mechanisms remain unclear, particularly because previous studies did not account for the recently identified obligatory β-subunits TMEM9A and TMEM9B, which regulate the trafficking and transport activity of ClC-3, ClC-4, and ClC-5.

**Results**

We expanded the clinical and genetic spectrum of *CLCN3* disease by identifying 15 additional patients with neurodevelopmental disorders and characterizing these and previously unstudied variants using electrophysiological and cell-biological approaches. Most variants were heterozygous, occurred de novo, and affected residues distributed throughout the protein. Developmental delay typically becomes apparent during infancy, with symptoms appearing at an average age of seven months. Common features included impaired speech development, ataxia, autistic traits, and epilepsy.

Functional analyses incorporating TMEM9 co-expression showed that most disease-causing variants confer a gain of function by weakening TMEM9-mediated inhibition. This likely enhances H^+^-driven Cl^−^ accumulation, causing osmotic swelling and the formation of enlarged lysosome-like vacuoles. Several variants affected residues within the PI(3,5)P_2_-binding pocket that stabilizes the interaction between TMEM9 and ClC-3. However, other variants reduced TMEM9-mediated inhibition despite being located far from this interface. These variants altered ClC-3 voltage-dependent gating even in the absence of TMEM9.

**Impact**

Our study shows that heterozygous *CLCN3* variants cause a toxic gain of function by reducing inhibition of the late-endosomal Cl^−^/H^+^ exchanger ClC-3 by TMEM9 β-subunits, resulting in a broad spectrum of neurodevelopmental disorders.

Importantly, reduced TMEM9-mediated inhibition is not limited to variants near the TMEM9/PI(3,5)P_2_ interface but also occurs with variants that alter ClC-3 gating. This suggests that gating-associated conformational changes influence the binding of TMEM9 and/or PI(3,5)P_2_.

These findings identify the ClC-3/TMEM9 complex as a potential therapeutic target. Although pharmacological inhibition of ClC-3 may benefit patients with ClC-3 hyperactivity, specific inhibitors are not yet available and would likely need to be administered early in disease progression.

## Introduction

The survival and function of cells critically depend on the endolysosomal system, which plays a key role in the uptake and processing of extracellular substances, trafficking of membrane proteins, degradation of intracellular organelles, metabolic signaling, and many other processes. Impaired endolysosomal function is particularly deleterious for the nervous system because adult neurons cannot dilute protein aggregates through cell division and depend on precise subcellular trafficking to maintain the highly specialized localization of essential proteins. For instance, lysosomal storage diseases, although affecting lysosomes throughout the body, often manifest with prominent neurological signs.

Ion transporters along the endocytic pathway maintain the appropriate environment for the activity of luminal enzymes and affect vesicle trafficking and maturation (Chadwick et al, 2021; Scott and Gruenberg, 2011). Vesicles in the endolysosomal pathway are progressively acidified by the vesicular H^+^-ATPase and reach a luminal pH of about 4.7 in lysosomes (Freeman et al, 2023). Vesicular 2Cl^−^/H^+^ exchangers of the CLC family (Jentsch and Pusch, 2018), ClC-3 to ClC-7, may provide a shunting conductance for vesicular acidification (Günther et al, 2003; Hara-Chikuma et al, 2005; Li et al, 2002; Novarino et al, 2010). In addition, these CLCs raise luminal Cl^−^ concentration, which by itself is critical for vesicle maturation and enzyme activity (Cigić and Pain, 1999; Weinert et al, 2010; Scott and Gruenberg, 2011; Chakraborty et al, 2017; Wu et al, 2023). The biological importance of endolysosomal CLC exchangers is evident from knockout (KO) mouse models for all endolysosomal CLCs (Jentsch and Pusch, 2018) and from more recent reports that rare genetic changes in endosomal ClC-3 (Duncan et al, 2021), ClC-4 (Veeramah et al, 2013; Palmer et al, 2018) and ClC-6 (Polovitskaya et al, 2020) are associated with a spectrum of human neuropsychiatric disorders.

The CLC gene family encodes both plasma membrane Cl^−^ channels and intracellular 2Cl^−^/H^+^ exchangers (Jentsch and Pusch, 2018). CLCs function as dimers, with each subunit possessing its own permeation pathway, which can be gated independently. Coordinated, voltage-dependent activation of both subunits—referred to as common gating—requires large conformational changes that include the cytosolic carboxy-termini (Duffield et al, 2003; Ludwig et al, 2013; Wan et al, 2024). While most CLCs function as homodimers, ClC-3 can associate with ClC-4 to form heterodimers (Suzuki et al, 2006; Guzman et al, 2017; Weinert et al, 2020).

The ClC-3 exchanger is found in all tissues, including neurons, where it localizes primarily to endosomes and less so to synaptic vesicles (Stobrawa et al, 2001; Li et al, 2002; Suzuki et al, 2006; Weinert et al, 2020). Its localization depends on alternative splicing, which alters its cytoplasmic amino-terminus. ClC-3a and ClC-3b reside in late endosomes, whereas ClC-3c is targeted to recycling endosomes and can partially reach the plasma membrane, where it is amenable to electrophysiological analysis (Guzman et al, 2015). Genetic ablation of *Clcn3* in mice caused profound neurodegeneration (Stobrawa et al, 2001). In humans, severe neurodevelopmental disorders were found in subjects carrying *CLCN3* variants, some of which exhibited loss or gain of transport function (Duncan et al, 2021).

Recently, the single transmembrane domain proteins TMEM9 (T9A) and TMEM9B (T9B) were identified as obligatory β-subunits of ClC-3, -4 and -5 (Planells-Cases et al, 2025). These subunits are crucial for protein stability, trafficking, and regulation of ion transport. Both subunits have similar roles as shown by functional assays and KO mice. A cytosolic phosphorylated acidic cluster (pAC) on TMEM9/9B (collectively referred to as T9) proteins reduces the plasma membrane expression of CLC/T9 complexes. Unlike other CLC β-subunits such as Ostm1 (Lange et al, 2006), barttin (Estévez et al, 2001) or GlialCAM (Jeworutzki et al, 2012), T9 proteins tonically inhibit CLC ion transport, with their C-terminal inhibitory domains (CID) blocking the cytoplasmic opening of the Cl^−^ pathway (Planells-Cases et al, 2025; Schrecker et al, 2025). Partial relief of this inhibition enables a large dynamic range of activation. The discovery of these obligatory subunits required a revision of previous analysis of disease-causing variants in *CLCN3* and *CLCN4*. Several pathogenic variants located close to the T9 binding site display a toxic gain of ion transport because they partially lost their sensitivity to T9-mediated inhibition (Planells-Cases et al, 2025).

Until now, only one homozygous and 10 heterozygous potentially disease-causing variants of *CLCN3* have been reported (Duncan et al, 2021; Nakashima et al, 2023). Common clinical findings included moderate to severe developmental delay, intellectual disability, structural brain abnormalities, behavioral disorders, hypotonia, seizures, and failure to thrive. Functional analysis had only been performed for a subset of variants (Duncan et al, 2021) and, with two exceptions (Planells-Cases et al, 2025), lacked co-expression of T9 subunits. The mechanisms by which these variants lead to disease remain largely unclear.

The aim of our study is to expand the genotypic and phenotypic spectrum of *CLCN3*-related neurodevelopmental disorders and to understand the biophysical and cellular mechanisms by which variants lead to pathology. We identified additional subjects carrying rare *CLCN3* variants, thereby expanding the known spectrum of *CLCN3*-related disorders. Functional analysis of these and previously published variants suggested that more than 65% of missense variants were hyperactive in the presence of the inhibitory T9A subunit, consistent with their pathogenicity in heterozygous subjects. Our study shows that even changes in residues not located close to the region that binds the inhibitory domain of T9A can partially relieve T9A-mediated inhibition and reveals a link between ClC-3 gating and T9-mediated inhibition.

## Results

### Cohort description

We newly describe 15 unrelated individuals with neurodevelopmental disorder (NDD) harboring *CLCN3* variants. With one exception (p.Thr570Ile), these variants have not been described before. Together with the previously published group of 11 individuals carrying 10 different variants (Duncan et al, 2021), the combined cohort of 26 individuals carried a total of 20 missense (one found in three subjects), 1 in-frame deletion of a single amino acid, 1 splice-site, and 1 truncating variant (found in two siblings (Duncan et al, 2021)). With one exception (p.Trp385Cys), the missense variants were found in a heterozygous state.

We focused clinical analysis on those 16/26 subjects whose variants resulted in functional changes of ClC-3 when analyzed in vitro, or on those carrying homozygous likely loss-of-function variants. Detailed clinical and genetic information for these 16 individuals, together with a short summary of our functional analysis, is provided in Dataset EV1 and in an abridged form in Table 1. Dataset EV2 gives a short summary of clinical features, in silico predictions and functional results for variants that appear normal in our functional assays.

**Table 1:** Genetic and clinical data of individuals carrying *CLCN3* missense variants that changed properties of ClC-3/T9A chloride/proton exchangers, as indicated, and of two frameshift and splice site variants.

Subject no. First reported	1 Present study	2 Duncan et al, 2021 (#10.1)	3 Present study	4 Duncan et al, 2021 (#1)	5 Present study	6 Present study	7 Present study	8 Duncan et al, 2021 (#2)
***CLCN3*** **variant information**								
**Genomic position (GRCh38)**	Chr4:g.169687675_169687678del	Chr4:g.170608819:AAAAG:A	Chr4:169697326 G > T	Chr4:g.169680143 A > G	Chr4:g.169680143 A > T	Chr4:g.169680148_169680150del	Chr4:g.169680149 A > T	Chr4:g.169692139 T > C
**c.DNA change (**NM_001829.4**)**	c.1017+5 G > A	c.336_339del	c.1155 G > T	c.254 A > G	c.254 A > T	c.259_261del	c.260 A > T	c.755 T > C
**Amino acid change**	p.?	p.Lys112AsnfsTer6	p.Trp385Cys	p.Tyr85Cys	p.Tyr85Phe	p.Asp87del	p.Asp87Val	p.Ile252Thr
**Inheritance**	Homozygous (parents unaffected)	Homozygous (parents unaffected)	Homozygous (parents unaffected)	De novo	De novo	De novo	De novo	De novo
**Detected by trio/singleton WES or panel**	Trio WES	Trio WGS	Trio WGS	Trio WES	Trio WGS	Trio WES	Single WGS	Trio WES
**Conclusion of functional testing**	N/A (likely LOF)	N/A (LOF)	Unclear	GOF	GOF	GOF	GOF	GOF
**Patient information**								
**Institution**	Tbilisi State Medical University, Georgia	University Hospital of Düsseldorf, Germany	University of Lakki Marwat Khyber Pakhtunkhwa, Pakistan	Erasmus University Medical Center, Netherlands	Peninsula Clinical Genetics, Royal Devon University Hospital Trust, UK	Children’s Clinical University Hospital, Riga, Latvia	Helios Klinikum Erfurt, Germany	Bambino Gesù Children’s Hospital, Italy
**Age at inclusion**	3 years	5 years 5 months	15 years	21 years	8 years	10 years	10 month	10 years
**Sex**	Female	Male	Female	Female	Female	Male	Male	Male
**Gestational age, weeks**	36	40	38	40	40	40	34	39
**Neonatal findings**	Hypotonia, nystagmus	Hypotonia, nystagmus	Hypotonia, dystonia, failure to thrive	Normal	Feeding difficulties	Normal	Hypotonia	NA
**Age at developmental concern**	2 months	1–2 months	15 months	before 12 months	NA	6 months	1 month	10 months
**Neonatal feeding difficulties**	Yes	No	Yes	No	Yes	No	Yes	No
**Neurodevelopment, cognition and behavior**								
**Speech development (age at first words/sentences)**	Absent	Absent	Normal (14/19 months)	Delayed	Delayed (18 months/NA)	Delayed (12 months/NA)	NA	Absent
**Gross motor development (age of sitting/walking)**	Not able to sit or walk	Not able to sit or walk	Ambulant (16/19 months)	Ambulant (delayed)	NA (NA/19 months)	Ambulant (12 months/3 years)	Not able at 10 months	Ambulant (15 months/6 years)
**Fine motor development**	Delayed	No hand function	Can hold a pen, eat alone	Delayed	NA	Delayed	NA	Delayed
**Developmental regression**	No	No	NA	No	No	Yes	NA	Yes
**DD/ID**	Severe/severe	Severe/severe	Mild/mild	Moderate/moderate	Moderate/NA	Severe/NA	NA	NA/severe
**Hypotonia central/limbs**	Yes/Yes	Yes/No	NA	No	NA	No	NA	Yes/Yes
**Autistic features**	No	No	Yes	Yes	NA	Yes	NA	Yes
**Mood or behavioral abnormalities**	No	No	Anxiety, self-damaging and aggressive behavior	ADHD, self-damaging behavior	NA	ADHD, self-damaging behavior	NA	No
**Sleep disorder**	Yes	Yes, severe sleep apnea syndrome	Narcolepsy	No	Yes	NA	NA	Yes
**Other movement disorder**	Spasticity in lower limbs	Bilateral spastic cerebral palsy GMFCS Level 5	Generalized dystonia	NA	No	No	NA	No
**Seizures**	Spasms, hypsarrhythmia	Focal seizure	Yes	No	No	No	Yes	No
**Brain MRI**	NA	Abnormal	NA	NA	Abnormal	Normal	Abnormal	NA
**Other clinical findings**								
**Vision/eye abnormalities**	Strabismus, nystagmus	Cortical visual impairment, strabismus, nystagmus	Strabismus, nystagmus	No	Cortical visual impairment, hypermetropia	Hypermetropia, astigmatism	NA	Strabismus, oculomotor apraxia
**Hearing loss**	No	No	No	No	NA	No	NA	No
**Other clinical information**	Frequent infections	Cryptorchidism, microdontia, gingival overgrowth, coliosis, acquired contractures, hip dislocation, frequent infections, hypothyroidism	Syndactyly of 2nd and 3rd toes of the right foot, vitiligo of hands. Died at age 15 years.	NR	Scoliosis	NR	NR	Phimosis, joint hypermobility, delayed eruption of teeth, partial growth hormone deficiency, one febrile seizure

Individual no. First reported	9 Present study	10 Duncan et al, 2021 (#3)	11 Present study	12 Present study	13 Duncan et al, 2021 (#7)	14 Present study	15 Duncan et al, 2021 (#8)	16 Present study
***CLCN3*** **variant information**								
**Genomic position (GRCh38)**	Chr4:g.169692243 C > T	Chr4:g.169695646 T > C	Chr4:g.169695655 G > T	Chr4:g.169704143 C > T	Chr4:g.169704143 C > T	Chr4:g.169706936 A > G	Chr4: 169706937 T > C	Chr4:g.169707245 A > G
**c.DNA change (**NM_001829.4**)**	c.859 C > T	c.971 T > C	c.980 G > T	c.1709 C > T	c.1709 C > T	c.1819A>G	c.1820T>C	c.2128 A > G
**Amino acid change**	p.His287Tyr	p.Val324Ala	p.Gly327Val	p.Thr570Ile	p.Thr570Ile	p.Ile607Val	p.Ile607Thr	p.Arg710Gly
**Inheritance**	NA, heterozygous	De novo	De novo	De novo	De novo	De novo	De novo	De novo
**Detected by trio/singleton WES or panel**	Duo WES	Trio WES	Trio WGS	Panel	Trio WES	Trio WES	Trio WES	Trio WES
**Conclusion of functional testing**	GOF	GOF	GOF	GOF	GOF	GOF	GOF	GOF
**Patient information**								
**Institution**	Centre for Genetics and Reference Centre for Developmental Anomalies and Malformative Syndromes, CHU Dijon, France	University of California, San Francisco, USA	Hospices Civils de Lyon, CHU de Clermont-Ferrand, France	Cincinnati Children’s Hospital, USA	University of British Columbia, Canada	Institute of Human Genetics, University of Bonn, Medical Faculty and University Hospital Bonn, Germany	University Medical Center Hamburg Eppendorf, Germany	Center of Medical Genetics, Antwerp University Hospital/ University of Antwerp; UZA/UA Center of Medical Genetics, Edegem, Belgium
**Age at inclusion**	13 years	17 years	25 years	NA	NA	4.5 years	23 days	2 years
**Sex**	Male	Male	Male	Female	Female	Male	Female	Male
**Gestational age, weeks**	41	39	41	41	40	38	39	40
**Neonatal findings**	Normal	Feeding difficulties, failure to thrive	Axial hypotonia, segmental hypertonia, poor visual contact	Hypotonia, feeding difficulties, failure to thrive	Normal	Hypotonia, feeding difficulties	Feeding difficulties, failure to thrive	Normal
**Age at developmental concern**	18 months	NA	Neonatal	1 week	6 months	3–4 months	NA	1 year
**Neonatal feeding difficulties**	No	Yes	No	Yes	No	Yes	Yes	No
**Neurodevelopment, cognition and behavior**								
**Speech development (age at first words/sentences)**	Delayed	Absent	Delayed (4 years/10 years)	Delayed	Normal	Delayed (18 months/4 years)	NA	Normal
**Gross motor development (age of sitting/walking)**	Ambulant (6/16 months)	Does not walk independently	Ambulant (18 months/5 years)	Ambulant (10/26 months)	Ambulant (9/30 months)	Walks little with support (14 months/3.5 years with support)	NA	Walks with support (8 months/26 months with support)
**Fine motor development**	Delayed, can point at an object	NA	Delayed	Delayed	Delayed	Delayed	NA	Delayed
**Developmental regression**	No	NA	No	Yes	No	No	NA	Yes
**DD/ID**	NA	NA/severe	Moderate/moderate	Severe/severe	Mild/moderate	Severe/NA	NA	Severe/No
**Hypotonia central/limbs**	No	Yes	No	Yes/Yes	Yes/Yes	Yes/Yes	No	Yes/Yes
**Autistic features**	Yes	No	No	Yes	No	Yes	NA	Yes
**Mood or behavioral abnormalities**	Anxiety, self-damaging behavior	NA	Anxiety	Anxiety, self-damaging, and aggressive behavior	ADHD, anxiety	Anxiety, self-damaging behavior	NA	No
**Sleep disorder**	Yes	NA	No	Yes, severe sleep apnea	No	No	NA	No
**Other movement disorder**	No	NA	Ataxia, extrapyramidal hypertonia, global akinesia	Ataxia, dystonia, tics, tremor	No	Ataxia, dystonia	NA	NA
**Seizures**	No	Myoclonus	Absence, myoclonus	Absence, drop seizures, gelastic	No	No	No	No
**Brain MRI**	NA	Abnormal	Abnormal	NA	Normal	NA	Abnormal	Abnormal
**Other clinical findings**								
**Vision/Eye abnormalities**		Optic dystrophy, retinal dystrophy, nystagmus	Myopia, photophobia	Hyperopia or myopia, pigment loss, strabismus	Anisometropia, strabismus	Strabismus	NA	No
**Hearing loss**	No	NA	Yes, sensorineural	Yes	No	No	NA	No
**Other clinical information**	Abnormal forefoot, angioma, hypopigmented patches	Chryptorchidism	Acquired contractures, stiff ankles, discrete equinus, clinodactyly of 5th finger, overlapping toes	Spasticity, small hands, microdontia, inverted nipples, autonomic dysfunction, reactive airways, frequent infections	Genu valgum, pronated feet, eczema, asthma	Joint hypermobility, stopic dermatitis, dysarthria	Arthrogryposis multiplex congenita. Deceased	Joint hypermobility, puffy dorsal side of hands and feet, problems with food texture

*GMFCS* gross motor function classification system, *GOF* gain of function, *het* heterozygous, *LOF* loss of function, *NA* not available, *N/A* not applicable, *No* feature not present, *NR* not reported, *WES* whole-exome sequencing, *WGS* whole-genome sequencing, *Yes* feature present.

Individuals 2, 4, 8, 10, 13, and 15 have been published before (Duncan et al, 2021), but lacked appropriate functional characterization of *CLCN3* variants. The clinical status of individuals #2, #4, #8, #10 and #13 has been updated since the first publication. Clinical details of the individual #15 are provided for comparison and have not been updated since the subject’s death occurred before the original report. Note that individual 2 had a severely affected sibling that was likewise homozygous for the frameshift variant (Duncan et al, 2021). This is an abridged version of Dataset EV1, which is available for download in Excel format.

**Figure 1:**
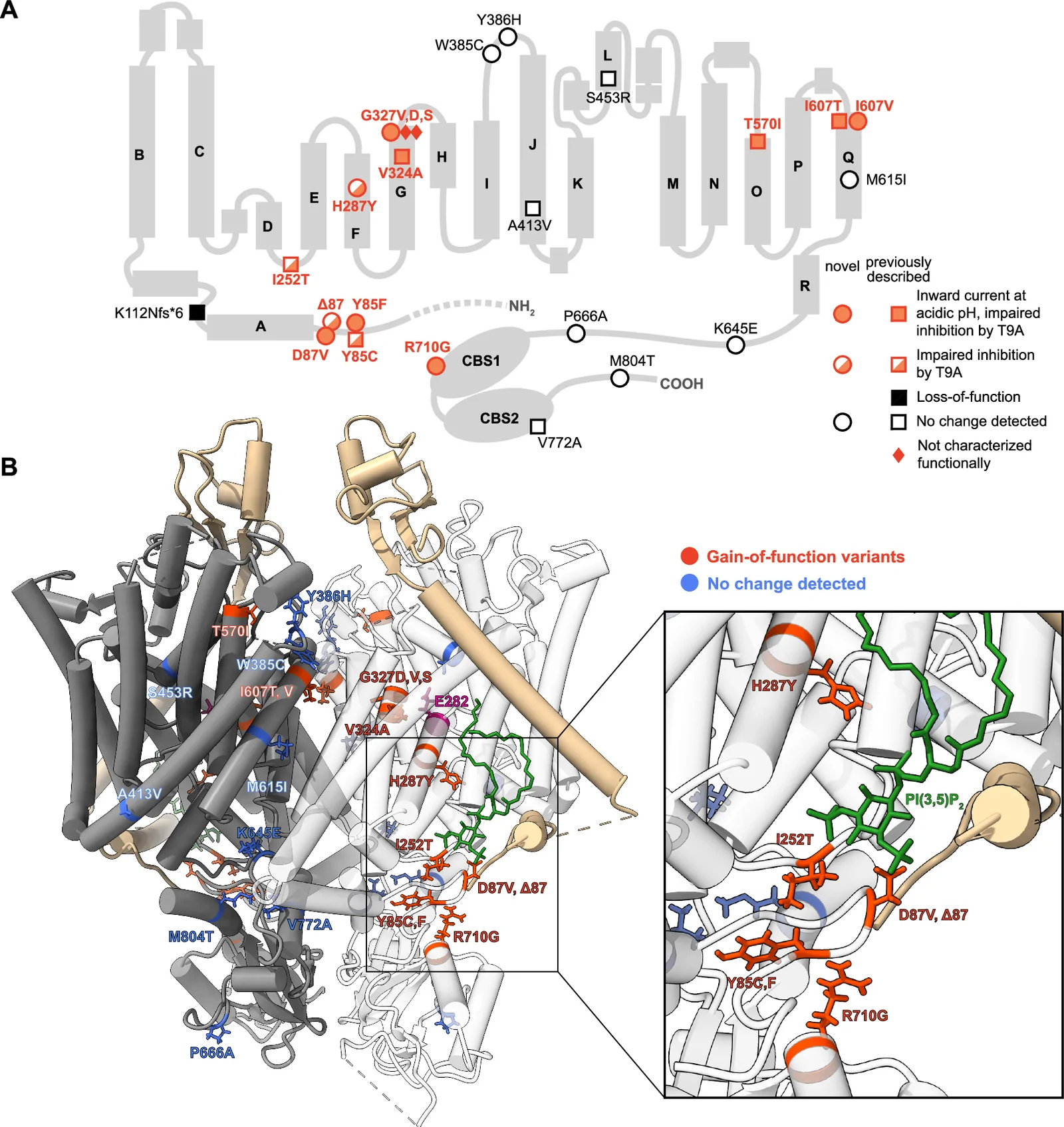
Positions in the ClC-3 protein of amino acids altered in subjects with NDD. (**A**) Schematic diagram of a ClC-3 monomer, depicting α-helices determined from cryo-EM structures as rectangles and cytoplasmic CBS (cystathionine β-synthase) domains as ovals. Functional characteristics of mutants coded by different symbols, with variants newly described here shown as circles. (**B**) Localization of these residues in the cryo-EM structure of ClC-3/T9A (PDB: 9DNX) (Schrecker et al, 2025). ClC-3 subunits shown in shades of gray, and T9A in beige. Individual ClC-3 monomers of the homodimer depicted in different shades. The box shows the magnification of the interface between ClC-3 and the CID of T9A, with residues altered in NDD subjects highlighted. Phosphatidylinositol-3,5-bisphosphate (PI(3,5)P_2_) (shown in green) is sandwiched between ClC-3 and the carboxyterminal inhibitory domain (CID) of T9A.

The 16 subjects carrying function-changing *CLCN3* variants (Table 1), of whom 6 had been reported previously (Duncan et al, 2021), were unrelated (7 females and 9 males). The median age at inclusion was 9 years (range 23 days to 25 years). Subject #1 carried a homozygous splice-site variant likely resulting in a loss-of-function (LOF), and subject #2 a biallelic variant predicting nonsense-mediated RNA decay and/or the production of a severely truncated, non-functional protein. One individual (#3) was homozygous for a missense variant leading to functional changes with unclear biological impact, whereas the remaining individuals carried heterozygous missense variants (or an in-frame deletion of a single amino), which led to a gain-of-function (GOF) in the functional assays described below. The heterozygous GOF variants were de novo (*n* = 12) or of unknown inheritance (*n* = 1). The variants, including those previously described (Duncan et al, 2021; Nakashima et al, 2023), affected cytoplasmic amino- and carboxy-termini, transmembrane helices and connecting loops of the encoded ClC-3 protein (Fig. 1A,B).

In silico predictions for the pathogenicity of these variants were obtained as detailed in Methods, with results given in Datasets EV1 and EV2. The mean CADD score across heterozygous variants with detected functional changes (Table 1 and Dataset EV1) was 28.6, ranging from 23.7 to 33.0, whereas CADD scores for the missense variants without detected functional alterations (Dataset EV2), ranged from 21.1 to 28.3. Two of the latter variants, but none of those listed as likely pathogenic (Datasets EV1 and EV2), occurred once in gnomAD v4.1.0.

### Clinical findings of subjects carrying variants that affect ClC-3 function

Clinical findings are summarized in Table 1 and Dataset EV1. Frequencies are reported as n/N, with N indicating the number of individuals for whom the respective feature was available. Among the 16 individuals carrying function-changing *CLCN3* variants or biallelic likely loss-of-function variants, the most frequent clinical hallmarks were developmental delay and/or intellectual disability (13/13, 100%), gross motor delay or impaired ambulation (13/14, 93%), intellectual disability (11/12, 92%), vision or eye abnormalities (11/13, 85%), language impairment (11/14, 79%), behavioral or psychiatric features (10/14, 71%), gastrointestinal or feeding symptoms (10/16, 63%), hypotonia (8/13, 62%), and autistic features (8/13, 62%).

Developmental concerns were recognized early, at a mean age of 6.7 months, ranging from the neonatal period to 18 months. Neonatal findings were present in 10/16 individuals (63%) and mainly included hypotonia (8/13, 62%), neonatal feeding difficulties (8/16, 50%), and failure to thrive (4/16, 25%). Where the distribution of hypotonia was specified, it involved both axial and limb musculature in 6/7 individuals and was restricted to central hypotonia in 1/7; one additional individual was reported as hypotonic without further specification.

Motor development was commonly delayed. Sitting age was reported for 9/16 individuals and ranged from 6 to 18 months. Independent walking, when achieved, occurred between 1.3 and 6 years. Two individuals walked only with support, and at least two remained non-ambulant beyond 3 years of age. Movement disorders were reported less consistently and included ataxia in 3/10 individuals (30%) and dystonia in 3/11 (27%). Additional isolated findings included extrapyramidal hypertonia with global akinesia, tics, tremor, lower-limb spasticity, and severe bilateral spastic cerebral palsy.

Cognitive and language impairment formed a central part of the phenotype. Developmental delay and/or intellectual disability were present in all individuals with available data (13/13, 100%). Intellectual disability was reported in 11/12 individuals (92%) and ranged from mild to severe among individuals aged 6 years or older. Formal neuropsychological testing was available for two individuals; in the remainder, the degree of impairment was based on clinical assessment. Language impairment was present in 11/14 individuals (79%). At last follow-up, 8/12 individuals with available information had some verbal communication, ranging from single words to fluent speech, whereas 4/12 remained non-verbal at last follow-up.

Behavioral and psychiatric manifestations were frequent (10/14, 71%). These included autistic features in 8/13 individuals (62%), self-damaging behavior in 6/13 (46%), anxiety in 6/11 (55%), ADHD in 3/10 (30%), and aggressive behavior in 2/13 (15%). Sleep disorders were reported in 7/12 individuals (58%), including severe sleep apnea in 2/12 and narcolepsy in 1/12. Developmental regression occurred in 4/12 individuals (33%) and affected language, motor function, cognition, and/or social skills. Of note, all these individuals displayed autistic features.

Epilepsy was present in 7/16 individuals (44%), with seizure onset ranging from the neonatal period to 6.5 years. Seizure type at onset was available for 5/7 individuals with epilepsy and included epileptic spasms, focal-onset hypermotor seizures, myoclonic seizures, absences, and unknown-onset seizures with impaired awareness. Two individuals developed additional seizure types during follow-up, and one experienced status epilepticus. At the last follow-up, 2/7 individuals with epilepsy were seizure-free, whereas 1/7 had treatment-resistant seizures.

Brain MRI was abnormal in most individuals for whom imaging was available. The most recurrent findings were hypoplasia or agenesis of the corpus callosum and pontine hypoplasia. Additional findings included cerebral atrophy, ventriculomegaly, abnormal white-matter signal, mild cerebellar tonsillar descent, prominent superior cerebellar peduncle, aqueductal stenosis with small vermis, enlarged perivascular spaces, and an arachnoid cyst.

Vision or eye abnormalities were reported in 11/13 individuals with available ophthalmological information (85%). Specific findings included strabismus in 7/11 individuals (64%), refractive errors in 5/13 (38%), nystagmus in 4/13 (31%), cortical visual impairment in 2/10 (20%), and retinal or optic abnormalities in 2/13 (15%). Oculomotor apraxia, pigment loss, and photophobia were each reported in one individual. Hearing loss was reported in 2/12 individuals (17%).

Gastrointestinal or feeding symptoms were present in 10/16 individuals (63%). These included neonatal feeding difficulties in 8/16 (50%), chronic constipation in 6/13 (46%), gastroesophageal reflux in 5/14 (36%), and need for a permanent feeding tube in 2/12 (17%). Pulmonary features were reported in 4/11 individuals (36%) and included recurrent infections in 3/11, asthma or reactive airways in 2/11, and severe sleep apnea in 2/11.

### Functional analysis of *CLCN3* variants

All but one variant affecting one amino acid were identified in the heterozygous state. Two of the three remaining homozygous variants affect either intronic sequences close to a splice site (#1) or lead to an early frameshift (#2, individual 10.1 reported by (Duncan et al, 2021)). These LOF variants cannot be tested functionally in our assays.

ClC-3 missense and in-frame deletion variants were characterized using electrophysiological recordings of plasma membrane currents and microscopy-based analysis of endolysosomal compartments. Both methods relied on ClC-3 overexpression in mammalian cells. Electrophysiological studies employed the ClC-3c splice variant, which partially localizes to the plasma membrane upon overexpression (Guzman et al, 2015). Vacuole formation was analyzed using ClC-3a, which targets late endosomes and induces enlarged vacuoles when not inhibited by T9A. This approach enables assessment of ClC-3 function in its native endosomal context and provides a sensitive assay for the modulation of ClC-3 activity by recently identified T9 β-subunits (Planells-Cases et al, 2025). These splice variants have identical ion transport properties when directed to the plasma membrane (Guzman et al, 2015). However, their localization to various membranes, which differ in lipid and phosphatidylinositide composition, may differentially affect their activity. In both assays, N-terminally Venus-tagged ClC-3 constructs were used to identify transfected cells. We also extended our analysis to previously described variants (Duncan et al, 2021) that lacked electrophysiological analysis or assessment of inhibition by T9 subunits.

**Figure 2:**
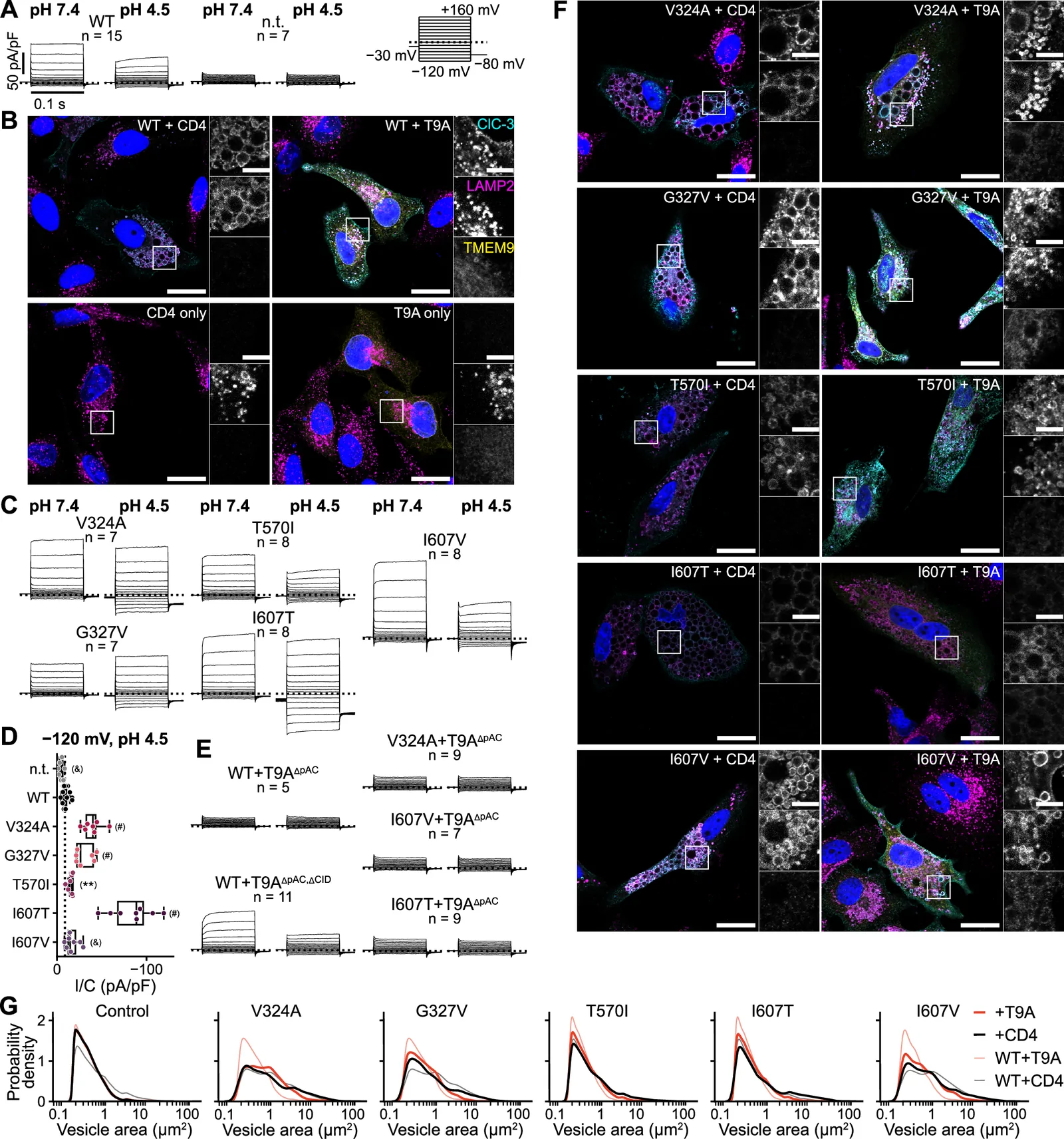
V324A, G327V, T570I, I607T, and I607V variants elicit inward currents at acidic bath pH and reduce sensitivity to T9A-mediated inhibition of vacuolization. (**A**) Averaged whole-cell current traces of non-transfected (n.t.) *TMEM206*^−/−^ HEK293 cells and cells transfected with wild-type Venus-ClC-3c (WT) elicited by the voltage step protocol shown on the top right obtained at bath pH 7.4 (left) and 4.5 (right). Dotted lines indicate zero current density or, in the protocol scheme, 0 mV. (**B**) Immunofluorescent staining of WT HeLa cells overexpressing T9A (right) or CD4 (left) as a control with or without WT Venus-ClC-3a (WT). Overexpressed ClC-3 is shown in cyan, native LAMP2 is shown in magenta, and overexpressed T9A is shown in yellow; nuclei stained with DAPI are shown in blue. T9A suppressed ClC-3-mediated enlargement of LAMP-2-positive compartments. (**C**) Averaged whole-cell current traces of V324A, G327V, T570I, I607T, and I607V variants obtained at bath pH 7.4 and 4.5 as in (**A**). Dotted lines indicate zero current density. (**D**) Current densities at −120 mV, bath pH 4.5 obtained at the end of each voltage step from the same recordings as averaged in panels (**A**) and (**C**). Boxes indicate the median and quartiles of the distribution. Circles correspond to individual cells. The dotted line indicates the median WT current density. **P = 0.0021; ^&^*P* = 0.0042; ^#^*P* = 2.3 × 10^−5^ (against WT, Mann–Whitney *U* test; false discovery rate was controlled using the Benjamini–Hochberg procedure). (**E**) Averaged whole-cell current traces of WT or mutant ClC-3 co-expressed with either T9^ΔpAC^ or T9^ΔpAC, ΔCID^, obtained as in (**A**). Note slowed voltage-dependent activation of currents with co-expression of T9^ΔpAC, ΔCID^, reminiscent of findings by (Festa et al, 2024), who may have inadvertently compromised CID by adding an epitope. (**F**) Immunofluorescent staining of WT HeLa cells overexpressing ClC-3 variants either with T9A (right) or CD4 (left) as a control, performed at least in triplicate. (**G**) Estimated area of Lysosensor-positive vesicles in HeLa cells overexpressing T9A (red) or CD4 (black) with ClC-3 variants or alone (control) pooled from at least three independent transfections. Transparent lines represent WT ClC-3 with T9A (light red) or CD4 (gray). (**A**, **C**, **D**, **E**) Recordings were obtained from n cells per variant, with *n* indicated in (**A**, **E**), across at least three independent transfections. (**B**, **F**) Scale bars on the merged panels, 20 μm; scale bars on the close-ups, 5 μm. Source data are available online for this figure.

Overexpression of WT ClC‑3c in *TMEM206*^−/−^ HEK293 cells—used to eliminate acid-activated ASOR currents (Ullrich et al, 2019)—produced outwardly rectifying currents (representing electrogenic 2Cl^−^/H^+^ exchange) with near‑instantaneous voltage-dependent activation at pH 7.4 (Fig. 2A). Switching bath pH to 4.5, close to late endosomal/lysosomal-like acidity, moderately suppressed WT current and induced slow activation at strongly cytosol‑positive potentials (Fig. 2A). Overexpression of ClC‑3a in HeLa cells enlarged LAMP2‑positive compartments, an effect suppressed by co‑expression of T9A (Fig. 2B) as described (Planells-Cases et al, 2025). Unspecific effects of T9A overexpression were excluded by co-transfection of CD4, an unrelated transmembrane protein.

**Figure EV1:**
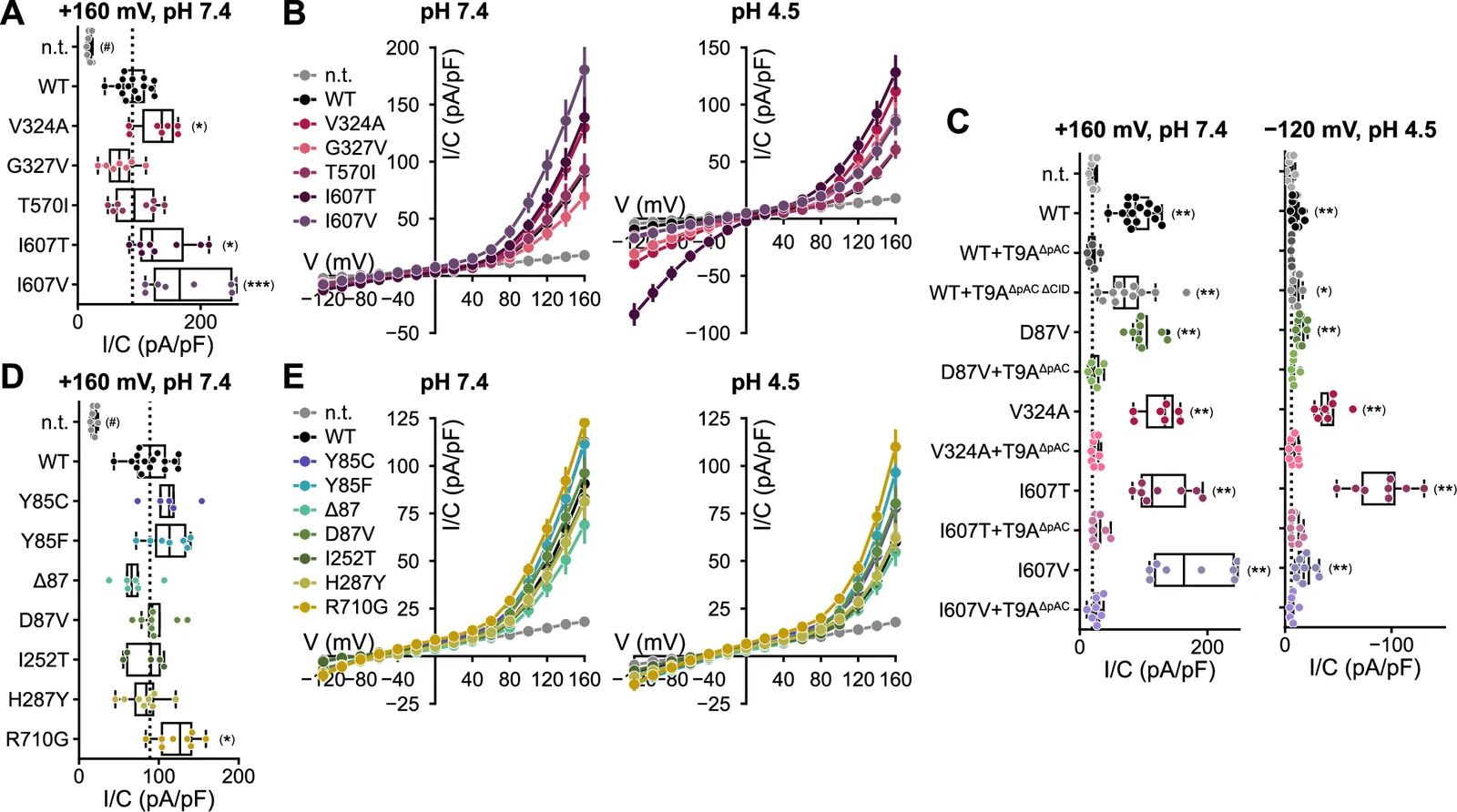
Detailed current parameters of variants found to be T9A-resistant in the vacuolization assay. Additional analysis of variants with large inward currents at acidic pH, shown in Fig. 2C,D, and variants with smaller plasma-membrane current changes but T9A-resistant vacuolization, shown in Fig. 3A, B. (**A**) Current densities at +160 mV, bath pH 7.4, for variants with large inward currents obtained from the same recordings as in Fig. 2C,D. (**B**) Current–voltage relationships for the variants shown in (**A**) at bath pH 7.4 (left) and pH 4.5 (right) from the same recordings as in Fig. 2C,D. (**C**) Current densities of WT or mutant ClC-3 expressed alone or with T9A^ΔpAC^ or T9A^ΔpAC,ΔCID^, measured at +160 mV, pH 7.4 (left) and −120 mV, pH 4.5 (right) obtained from the same recordings as in Figs. 2C,E and 3A. (**D**) Current densities at +160 mV, bath pH 7.4, for variants with small plasma-membrane current changes obtained from the same recordings as in Fig. 3A,B. (**E**) Current–voltage relationships for the variants shown in (**D**) at bath pH 7.4 (left) and pH 4.5 (right). Boxes indicate medians and quartiles; dotted lines indicate the median WT current density in (**A**) and (**D**), or in (**C**), the median WT + T9A^ΔpAC^ current density. In (**B**, **E**), error bars, SEM. In (**A**, **D**), WT and n.t. data are reproduced from Fig 2D for comparison; *P < 0.05; ***P < 0.001; ^#^*P* < 0.0001 against WT; in (**C**), **P < 0.01 against WT + T9A^ΔpAC^ (Mann–Whitney *U* test; false discovery rate was controlled using the Benjamini–Hochberg procedure).

Several variants generated substantial inward currents at acidic extracellular pH (Figs. 2C,D and EV1A,B), a property not observed in WT ClC-3 but previously reported for p.Ile607Thr (Duncan et al, 2021). p.Ile607Val, affecting the same residue in helix Q (Fig. 1A), as well as p.Val324Ala, previously described but uncharacterized (Duncan et al, 2021), and newly identified p.Gly327Val, likewise generated such inward currents. Both Val^324^ and Gly^327^ are located on helix G (Fig. 1A). Of note, substitutions of the same glycine (p.Gly327Ser and p.Gly327Asp) were previously found as de novo variants in two subjects with severe neurological disease (Nakashima et al, 2023). p.Ile607Val (Fig. 2C) activated more slowly at positive potentials, but the relevance of this feature for pathogenicity remains unclear.

The appearance of 2Cl^−^/H^+^ exchange currents at negative potentials may represent a toxic gain of function. In vivo, however, all endosomal CLCs associate with obligatory T9 β-subunits that strongly inhibit their ion transport (Planells-Cases et al, 2025). It is therefore crucial to determine whether these additional currents escape T9-mediated inhibition. Because T9 proteins suppress ClC-3 PM currents both by blocking the Cl^−^ pathway and by reducing surface expression (Planells-Cases et al, 2025; Schrecker et al, 2025), we co-expressed ClC-3 variants with T9A^ΔpAC^, a mutant lacking an internalization signal that traffics to the surface together with the CLC protein. Indicating potent T9 inhibition, resulting currents from three variants (p.Ile607Val, p.Ile607Thr, and p.Val324Ala) were indistinguishable from background (Figs. 2E and EV1C). This was not observed with a control construct (T9A^ΔpAC,ΔCID^) (Planells-Cases et al, 2025) with additional disruption of the C-terminal inhibitory domain CID (Figs. 2E and EV1C). In the vacuolization assay, which reflects cumulative ClC-3 activity overexpression time (Planells-Cases et al, 2025), however, vacuole generation by these variants was resistant to WT T9A (Fig. 2F,G), although the altered residues cannot directly interact with T9’s CID because they are located within the transmembrane region (Planells-Cases et al, 2025; Schrecker et al, 2025) (Fig. 1). Hence, although largely inhibited by T9 at the plasma membrane, these variants exert a toxic gain of function in lysosomes.

**Figure 3:**
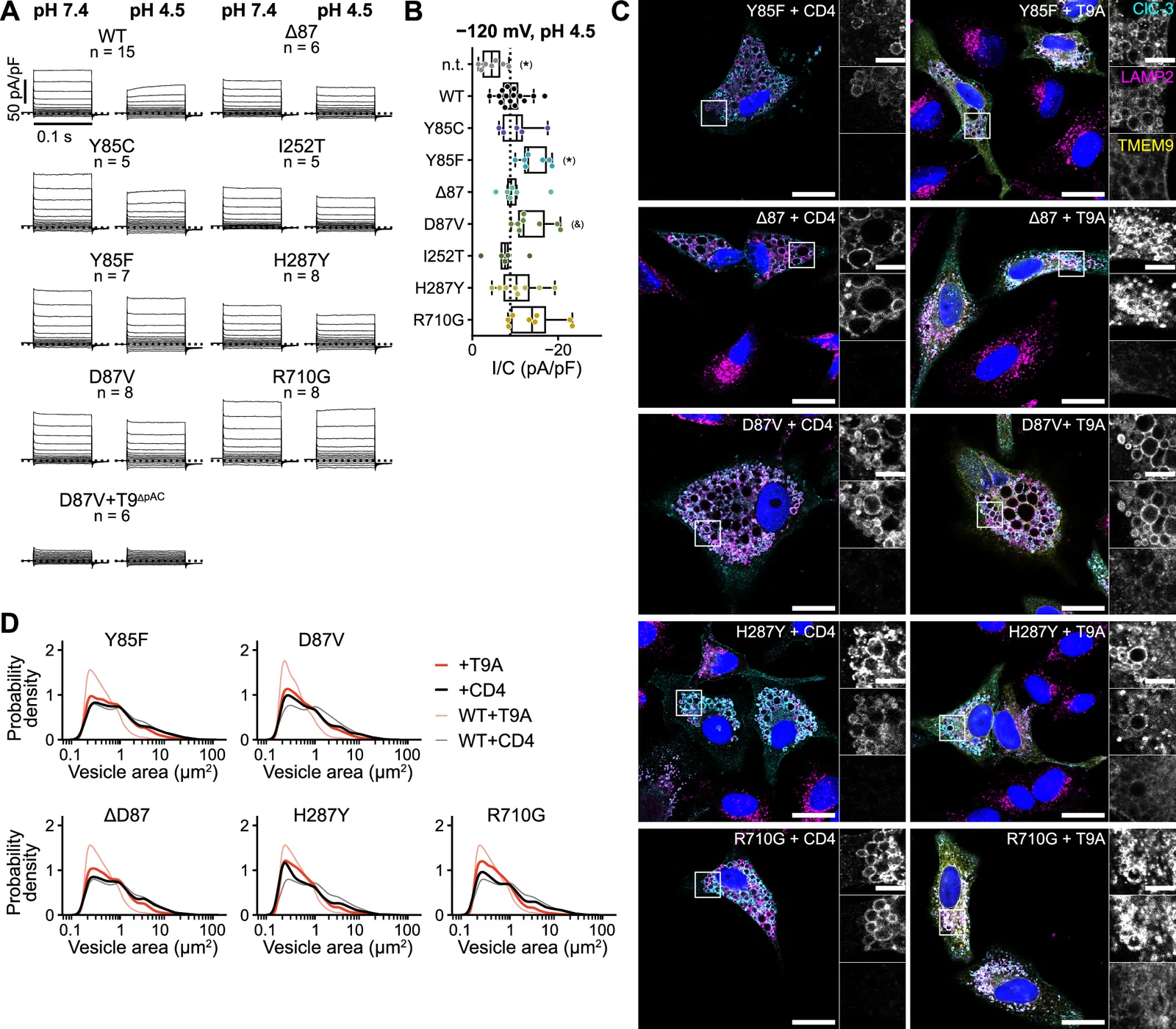
Y85C, Y85F, Δ87, D87V, I252T, H287Y, and R710G abolish sensitivity to T9A-mediated inhibition and mildly affect ClC-3-mediated PM currents. (**A**) Averaged whole-cell current traces of p.Tyr85Cys, p.Tyr85Phe, p.Asp87del, p.Asp87Val, p.Ile252Thr, p.His287Tyr, and p.Arg710Gly variants obtained at bath pH 7.4 (left) and 4.5 (right) as in Fig. 2A. WT traces are reproduced from Fig. 2A for comparison. Dotted lines indicate zero current density. (**B**) Current densities at −120 mV, bath pH 4.5, from the same recordings shown in (**A**). Circles correspond to individual cells. Recordings were obtained from n cells per variant, with n indicated in (**A**), across at least three independent transfections. Boxes indicate the median and quartiles of the distribution. Dotted line indicates the median WT current density. WT and n.t. data are reproduced from Fig. 2D for comparison. ^&^*P* = 0.035; *P = 0.015 (against WT, Mann–Whitney *U* test; false discovery rate was controlled using the Benjamini–Hochberg procedure). (**C**) Immunofluorescent staining of cells overexpressing ClC-3 variants with T9A (right) or CD4 (left). Scale bars on the merged panels, 20 μm; scale bars on the close-ups, 5 μm. (**D**) Estimated area of Lysosensor-positive vesicles in HeLa cells overexpressing T9A (red) or CD4 (black) with ClC-3 variants or alone (control). Transparent lines represent WT ClC-3 with T9A (light red) or CD4 (gray). Source data are available online for this figure.

**Figure 4:**
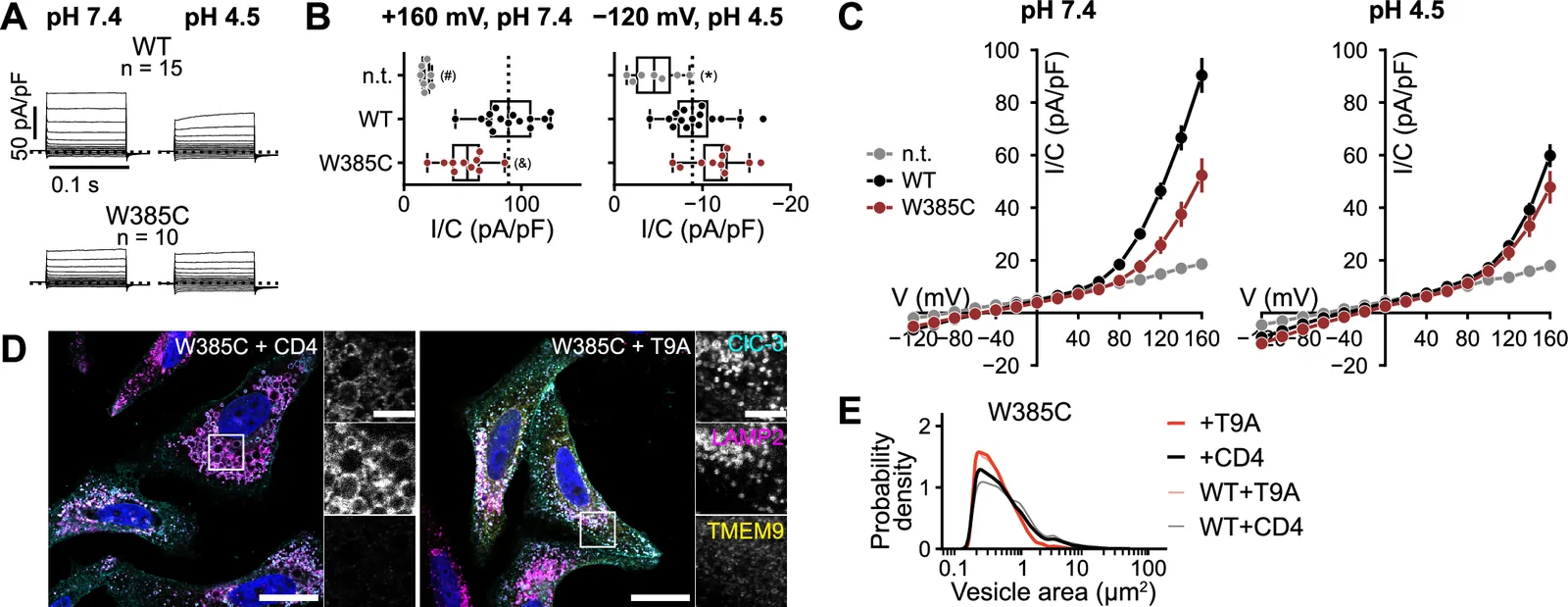
The homozygous p.Trp385Cys variant shows less inhibition by acidic external/luminal pH and retains sensitivity to inhibition by T9A. (**A**) Averaged whole-cell current traces of cells transfected with wild-type Venus-ClC-3c (WT) or W385C variant (which was found in a homozygous state) elicited by the voltage step protocol shown on the top right obtained at bath pH 7.4 (left) and 4.5 (right). Dotted lines indicate zero current density. WT data is reproduced from Fig. 2A for comparison. (**B**) Current densities at +160 mV, bath pH 7.4 (left) and −120 mV, bath pH 4.5 (right). Boxes indicate the median and quartiles of the distribution. Circles correspond to individual cells. The dotted line indicates the median WT current density. (**C**) Current–voltage relationship curves derived from the currents at the end of each 100-ms voltage step at bath pH 7.4 (left) and 4.5 (right) obtained from the same recordings as in (**A**). Error bars, SEM. *P = 0.008; ^&^*P* = 5.2 × 10^−4^; ^#^*P* = 2.3 × 10^−5^ (against WT, Mann–Whitney *U* test; false discovery rate was controlled using the Benjamini–Hochberg procedure). Recordings were obtained from n cells per variant, with *n* indicated in (**A**), across at least three independent transfections. (**D**) Immunofluorescent staining of cells overexpressing ClC-3^W385C^ with T9A (right) or CD4 (left). Scale bars on the merged panels, 20 μm; scale bars on the close-ups, 5 μm. (**E**) Estimated area of Lysosensor-positive vesicles in HeLa cells overexpressing the p.Trp385Cys variant with T9A (red) or CD4 (black). Transparent lines represent WT ClC-3 with T9A (light red) or CD4 (gray). Source data are available online for this figure.

Five novel variants in the N-terminus (p.Tyr85Phe, p.Asp87del, p.Asp87Val), helix F (p.His287Tyr), and the cytosolic CBS domain 1 (p.Arg710Gly), along with previously reported N-terminal p.Tyr85Cys and p.Ile252Thr in the D/E linker (Duncan et al, 2021) (Fig. 1), had minimal effects on ClC‑3-mediated PM currents in the absence of T9A. Only occasionally low-amplitude inward currents were observed at negative voltages (Figs. 3A,B and EV1E). However, immunofluorescence revealed robust vesicle enlargement in the presence of T9A ((Planells-Cases et al, 2025) and Fig. 3C,D). All residues except His^287^ are known to influence T9-mediated transport inhibition and participate in binding the CID of T9 (Planells-Cases et al, 2025; Schrecker et al, 2025). His^287^ appears consistent with this mechanism as well—its location at the cytosolic end of helix F positions it close to the cytosolic opening of the chloride permeation pathway blocked by CID (Schrecker et al, 2025) (Fig. 1B). Despite inducing vacuolization with T9A overexpression (Fig. 3C,D), these variants did not elicit PM currents when co-expressed with T9A^ΔpAC^ as exemplified by p.Asp87Val (Figs. 3A and EV1C). Collectively, these variants produce a toxic gain of transport with a mechanism now well understood.

p.Trp385Cys, the only homozygous missense variant in our cohort, produced smaller plasma membrane currents when studied in ClC-3c (Fig. 4A–C). While this is compatible with a partial loss of function, its insensitivity to external acidification could instead lead to increased activity in endolysosomes. Since T9 co-expression fully suppressed vacuole enlargement (Fig. 4D,E), the pathogenicity of this variant remains unclear.

**Figure EV2:**
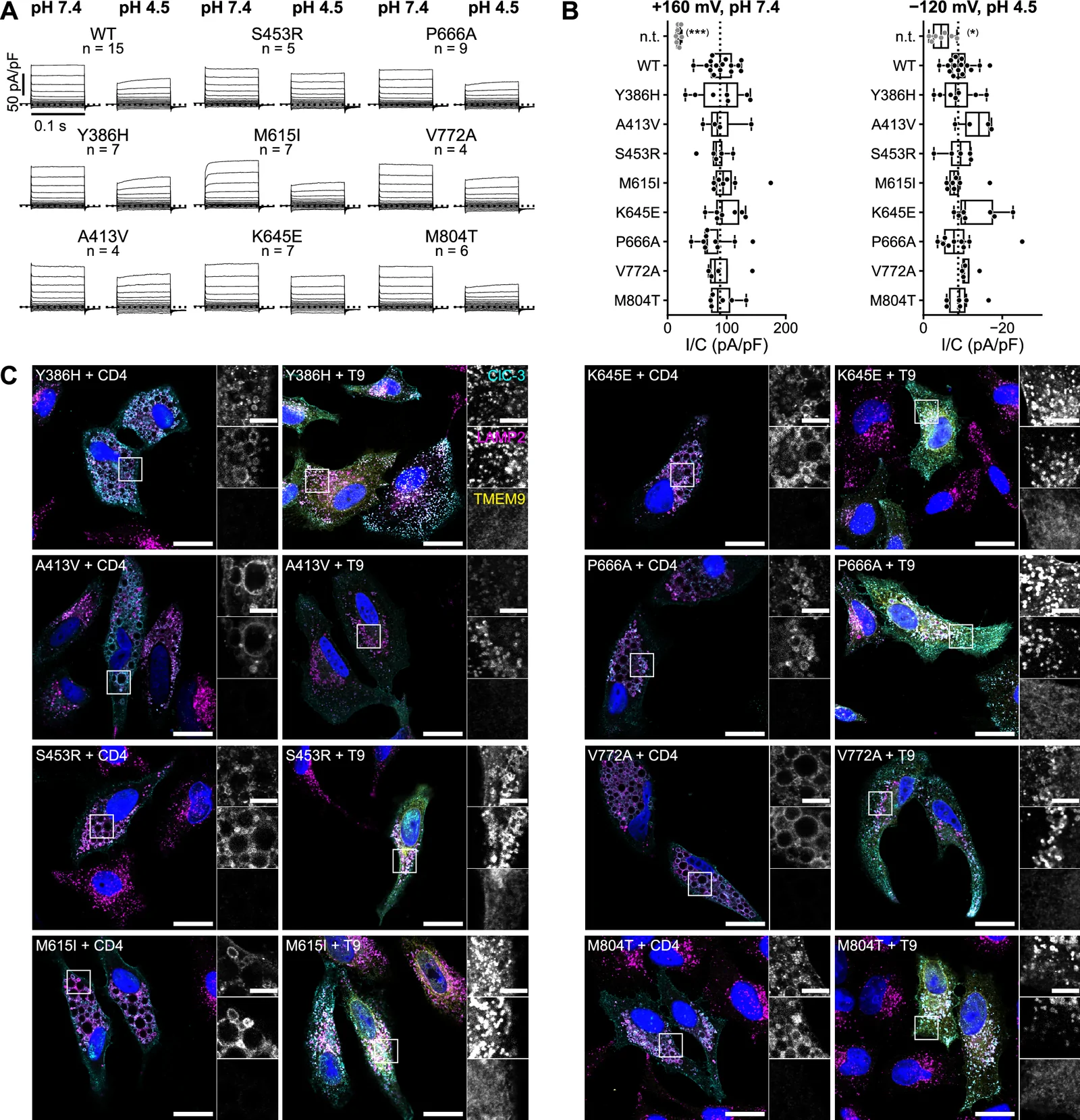
Variants lacking functional effects in our assays. p.Tyr386His, p.Ala413Val, p.Ser453Arg, p.Met615Ile, p.Met604Thr, p.Lys645Glu, p.Pro666Ala, p.Val772Ala, and p.Met804Thr retain sensitivity to T9A-mediated inhibition and do not elicit significant inward currents at acidic bath pH. (**A**) Averaged whole-cell current traces of ClC-3 variants obtained at bath pH 7.4 (left) and 4.5 (right) as in Fig. 2A, WT data are reproduced from the same panel for comparison. Dotted lines indicate zero current density. (**B**) Current densities at +160 mV, bath pH 7.4 (left) and −120 mV, bath pH 4.5 (right). Boxes indicate the median and quartiles of the distribution. The dotted line indicates the median WT current density. WT and n.t. data are reproduced from Fig. 2C for comparison. Recordings were obtained from n cells per variant, with *n* indicated in (**A**), across at least three independent transfections. *P < 0.05; ***P < 0.001 (against WT, Mann–Whitney *U* test; false discovery rate was controlled using the Benjamini–Hochberg procedure). (**C**) Immunofluorescent staining of cells overexpressing ClC-3 variants with T9A (right) or CD4 (left). Scale bars on the merged panels, 20 μm; scale bars on the close-ups, 5 μm. *CLCN3* variants p.Ala413Val, p.Ser453Arg, and p.Val772Ala and associated clinical symptoms have been described before (Duncan et al, 2021), whereas the other variants (p.Tyr386His, p.Met615Ile, p.Lys645Glu, p.Pro666Ala, p.Met804Thr) are newly described here.

Except for minor changes in activation kinetics and pH sensitivity, no significant functional changes could be detected with currents of p.Tyr386His, pAla413Val, p.Ser453Arg, p.Met615Ile, p.Lys645Glu, p.Pro666Ala, p.Val772Ala, and p.Met804Thr variants (Fig. EV2A,B). These variants neither appreciably interfered with T9A-mediated inhibition in the vacuolization assay (Fig. EV2C). While these results do not support pathogenicity, it remains possible that our assays missed functionally relevant interaction partners or physiological conditions present in vivo.

In total, 12 of the 21 heterozygous variants affecting a single amino acid exhibited a bona fide GOF, consistent with a dominant toxic effect. The most notable alteration was resistance to T9A-mediated inhibition in the sensitive vacuolization assay, which did not strictly correlate with the proximity of the mutated residue to the CID-binding pocket.

## Discussion

### Both loss- and gain-of-function of ClC-3 can cause human disease

Loss-of-function (LOF) variants of *CLCN3* can lead to human neurodegenerative/developmental disease, as evident from the homozygous p.Lys112Asnfs*6 variant that truncates the ClC-3 protein before the first transmembrane domain (Duncan et al, 2021). It was found in two siblings, one of whom was followed up clinically in the present study (#2). Subject #1, who harbored a homozygous splice-site variant likely also leading to LOF, now adds a similar case. In mice, KO of ClC-3 entails severe neurodegeneration, which eventually results in a loss of the hippocampus (Stobrawa et al, 2001; Yoshikawa et al, 2002). Interestingly, similarly severe neurodegeneration is observed when only one aspect of ClC-3 function, the exchange of chloride for protons, but not chloride permeation, is eliminated in *Clcn3*^unc/unc^ mice by a single point mutation—but only when studied in a *Clcn4*^−^^/^^−^ background (Weinert et al, 2020). This is because ClC-3 and ClC-4, which in the brain mostly occur as heterodimers, can largely substitute for each other functionally (Weinert et al, 2020). Notably, the trafficking and stability of ClC-4 depend on the presence of ClC-3. In *Clcn3*^−/−^ mice, levels of ClC-4 proteins are markedly decreased, further potentiating the insult caused by the lack of ClC-3 (Weinert et al, 2020). We expect the same for subjects #1 and #2.

Of note, the pathology caused by the loss of ClC-3 cannot be attributed entirely to the resulting loss of ClC-4, as demonstrated by the pathology of *Clcn3*^td/td^ mice with disrupted ClC-3 ion transport, but maintained stabilization of ClC-4 (Balduin et al, 2025). Conversely, disruption of ClC-4, which does not affect ClC-3 levels, can lead to pathology (Palmer et al, 2018), although it is milder than with ClC-3 LOF, which goes hand in hand with a loss of ClC-4. Hence, the ability of ClC-3 and ClC-4 to substitute for each other functionally is not complete.

The heterozygous parents of LOF variants carrying #1 and #2 were unaffected, agreeing with the absence of pathology in heterozygous *Clcn3*^+/−^ mice (Stobrawa et al, 2001). We expect that all LOF variants of *CLCN3* will be tolerated in a heterozygous state and hence are rarely found in patients. This contrasts with *CLCN4* for which many pathogenic LOF variants were described (Palmer et al, 2018, 2023). This is due to the X-chromosomal localization of human *CLCN4:* males have no WT allele, which might compensate for the LOF variant.

Based on these considerations, it is not surprising that the majority of *CLCN3* variants in our cohort likely cause a gain-of-function (GOF). Variants increasing ClC-3 ion exchange activity, either quantitatively or qualitatively (e.g., by broadening the voltage range over which ion transport occurs), may exert a dominant toxic effect in heterozygous individuals. Our previous work (Duncan et al, 2021), which ignored the effects of T9 subunits, suggested p.Ile607Thr as a GOF variant. It moderately increased ClC-3 current at neutral pH. More importantly, it enabled currents at more negative cytoplasmic voltages, suggesting that its ion transport may be overstimulated at physiological endosomal voltages. In the present cohort, we identified an additional missense variant affecting the same residue, p.Ile607Val, as well as multiple other variants located in different regions of the protein that produced similar biophysical alterations. Together, these variants point to a shared pathogenic mechanism.

The recent discovery of T9 proteins as inhibitory subunits for the ClC-3 to ClC-5 clade (Planells-Cases et al, 2025) raised the question of whether these variants might be resistant to T9-mediated inhibition, a prerequisite for changes in voltage-dependence to have an effect. Whereas co-expression with T9A^ΔpAC^, which permits surface expression while retaining the inhibitory CID domain, suppressed these currents (Fig. 2E), the variants displayed sufficient resistance to inhibition to generate large vesicles in the vacuolization assay (Fig. 2F). This apparent discrepancy echoes earlier findings (Planells-Cases et al, 2025) showing that disease-causing variants may entail partial resistance to T9 in ClC-3-based vacuolization assays, while not generating detectable PM currents. This suggests that even a small relief from T9 inhibition can cause vacuolization, although it is not sufficient to increase plasma membrane currents above background. The apparently higher sensitivity of the vacuolization assay may be owed to the integration of small increases in volume over time, or to differences in the membrane environment. Both types of assays only qualitatively reflect ion transport, as the human variants may also affect subcellular trafficking of the transporters. The weakened T9-mediated inhibition of this class of variants, which display significant inward currents, raises the question of whether their altered current rectification is required for vacuolization and pathogenicity. Supporting this idea, disease-causing variants in ClC-6 (Polovitskaya et al, 2020; Zhang et al, 2023) and ClC-7 (Nicoli et al, 2019; Polovitskaya et al, 2024; Lee et al, 2024)—CLC exchangers without known inhibitory β-subunits—shift voltage dependence toward more negative voltages and cause vacuolization.

Another class of pathogenic *CLCN3* variants partially relieves T9-mediated inhibition without significantly changing PM currents in the absence of T9. The affected residues (Tyr^85^, Asp^87^, Ile^252^, His^287^, and Arg^710^) are located in a region previously shown to be important for binding the inhibitory carboxy-terminus (CID) of T9 proteins (Planells-Cases et al, 2025; Schrecker et al, 2025). Underlining shared pathogenic mechanisms, the ClC-4 equivalent of ClC-3 Asp^87^—deleted in subject #6 and replaced by Val in subject #7—was changed to Glu in an individual carrying the *CLCN4* p.Asp29Glu variant, and the pathogenic *CLCN4* p.Arg652Thr variant affects the residue corresponding to Arg^710^ in ClC-3 that is changed in the present *CLCN3* p.Arg710Gly variant (Palmer et al, 2023; Planells-Cases et al, 2025). These variants display a clear quantitative gain of transport activity without altering the biophysical properties of ClC-3 currents.

One missense variant (p.Trp385Cys) was identified in the homozygous state, with both parents being unaffected. Bioinformatic analyses suggested this variant to be damaging (Dataset EV1). Functionally, it showed features suggestive of increased endosomal activity, including reduced inhibition by acidic pH; however, it did not induce vacuolization upon overexpression together with T9A. Although these subtle functional alterations may be amplified by homozygous expression in the proband, it remains unclear whether they are sufficient to account for the observed disease phenotype.

Eight missense variants found in NDD individuals (Dataset EV2) neither significantly changed plasma-membrane currents nor showed resistance to T9 inhibition in the vacuolization assay (Fig. EV2). Our functional assays therefore failed to support their pathogenicity. However, these variants are extremely rare in or absent from population databases, and we cannot rule out that they may cause defects not captured by our overexpression assays, such as altered subcellular localization in neurons or changes in regulatory mechanisms.

In our assays, GOF variants caused the formation of large lysosome-like compartments, likely due to H⁺-gradient-driven luminal Cl⁻ accumulation, followed by osmotic swelling and impaired vesicle scission (Polovitskaya et al, 2020). It remains unclear whether this effect occurs only upon overexpression in vitro or also in human subjects, as observed with strongly activating *CLCN7* variants associated with vacuolization in many tissues (HOD syndrome, OMIM: 618541) (Leray et al, 2022; Nicoli et al, 2019; Polovitskaya et al, 2024). The absence of organomegaly, a hallmark of HOD, argues against this possibility in the present subjects. The precise mechanisms by which *CLCN3* variants lead to disease remain unclear and may vary with the particular type of variant. The observation that both LOF and GOF variants in *CLCN3* and *CLCN4* (Palmer et al, 2023; Planells-Cases et al, 2025) can produce similar symptoms suggests somewhat general effects, potentially including altered trafficking of neuronal receptors and channels (Qi et al, 2025).

### Implication for the structure–function relationship of ClC-3/T9

Several newly identified variants affected residues that are located in the region of ClC-3 that binds T9’s inhibitory carboxyterminal domain (CID). These findings confirm and extend our previous analysis (Planells-Cases et al, 2025; Schrecker et al, 2025) and provide a clear mechanism for a toxic GOF. Other variants affecting residues in different regions allowed inward currents at acidic pH, suggesting a gain of function. Intriguingly, all these variants also weakened T9-mediated inhibition in the vacuolization assay despite the fact that the altered residues are far from the CID-binding region. Hence, gating-associated conformational changes may change binding affinity. Notably, functional assays of disease-associated Arg^710^ and Thr^713^ variants (the equivalent residues of ClC-4 are found mutated in NDD (Planells-Cases et al, 2025); and Fig. 3) suggest that CID interacts with the cytoplasmic CBS1 domain even though both residues are not in close proximity to CID in the state captured by cryo-EM (Schrecker et al, 2025). Cytoplasmic CBS domains of CLC proteins undergo substantial, poorly defined rearrangements during ‘common’ gating of CLCs (Bykova et al, 2006; Leisle et al, 2020; Hilton et al, 2025) and hence might contact CID in a different conformational state. Intriguingly, alteration of residues involved in CID binding and T9-mediated inhibition (Planells-Cases et al, 2025; Schrecker et al, 2025), including the aforementioned Arg^710^ of CBS1 or N-terminal Tyr^85^ and Asp^87^, resulted in a slight increase of inward currents at acidic pH (Fig. 3A,B). Thus, modulation of T9 binding through gating-dependent conformational changes appears plausible.

The structural basis for CLC exchanger gating, which involves both subunits in a common process (Ludwig et al, 2013), remains poorly understood. The present work provides additional insights. ClC-3 Ile^607^, whose alteration to Thr (Duncan et al, 2021) or Val (this work) yields additional inward currents, resides at the interface between the two CLC monomers (Fig. 1B). In *E.coli* ecClC-1, replacing the equivalent Ile^442^ with Trp resulted in a predominantly monomeric transporter (Robertson et al, 2010). However, since overall gating is conserved with p.Ile607Thr and p.Ile607Val substitutions, these variants are unlikely to impede dimerization. Instead, they may subtly change subunit interactions that are necessary for common gating. The other two residues associated with inward currents, Val^324^ and Gly^327^, are located around the ‘external’ conserved Phe^326^, which interacts with the protonated E_gate_ in the course of the transport cycle (Leisle et al, 2020) (Fig. 1). Changing this ‘gating glutamate’ affects common gating of CLC channels and transporters, protopore gating of ClC-0, and exchange stoichiometry (Leisle et al, 2020; Palmer et al, 2023). It is conceivable that changes in the neighboring residues may perturb the movement of E_gate_ in the course of common gating.

Although the present cohort, which includes previously reported individuals (Duncan et al, 2021), is the largest published to date, it remains difficult to establish clear correlations between specific variants and clinical phenotypes. Only a general picture emerges: Homozygous LOF variants are associated with earlier onset and more severe symptoms compared to the heterozygous GOF variants. This included neonatal onset hypotonia and nystagmus, with seizures beginning at a mean age of 4 months and initially presenting as focal-onset seizures, epileptic spasms, or hypermotor seizures. Motor outcomes included bilateral spastic cerebral palsy in one subject, while dystonia was absent. Subjects with heterozygous GOF variants showed later developmental concerns (mean 7.7 months), no neonatal nystagmus, and later-onset seizures (mean 2.9 years), initially manifesting as generalized seizures such as absence seizures. Movement abnormalities included dystonia in three individuals, whereas bilateral spastic cerebral palsy was not observed.

### Conclusion

In conclusion, we report sixteen individuals with neurodevelopmental disorder (NDD) and function-changing *CLCN3* variants, of which six were previously reported: two with biallelic LOF variants, thirteen with heterozygous GOF variants, and one with a biallelic variant of unclear functional consequences. Although this is the largest known dataset, the sample size still limits the ability to establish robust genotype–phenotype correlations or to draw definitive conclusions regarding variant-specific clinical trajectories. In contrast to the previous studies (Duncan et al, 2021; Nakashima et al, 2023), our functional analysis systematically addressed the effects of recently identified inhibitory T9 β-subunits (Planells-Cases et al, 2025). Although relying on overexpression in mammalian cells and neglecting the effects of ClC-4 with which ClC-3 heteromerizes in the brain (Weinert et al, 2020; Balduin et al, 2025), our results revealed that ~2/3 of ClC-3 missense variants identified in subjects with NDD exhibit a gain of ion transport. These variants fell into two groups: those that reduce T9-mediated inhibition by altering residues within the binding region for its C-terminal inhibitory domain, and those changing ClC-3 gating, enabling currents across a broader voltage range. Notably, gating-altering variants also attenuated T9 inhibition, revealing a previously unrecognized link between gating-associated conformational changes and CID-binding affinity. Our findings advance clinical interpretation and mechanistic understanding of CLC/T9 ion exchangers.

## Methods

**Table:** Reagents and tools table

Reagent/resource	Reference or source	Identifier or catalog number
**Experimental models**		
WT HeLa cells	Leibniz-Institut DSMZ- Deutsche Sammlung von Mikroorganismen und Zellkulturen GmbH, Germany	ACC 57
TMEM206^−/−^ HEK293 cells	Ullrich et al, 2019	Clone 2E5
**Recombinant DNA**		
Venus-ClC-3c, mouse splice variant c	This study; pEGFP-C1 backbone from Clontech	
Venus-ClC-3a, human splice variant a	Planells-Cases et al, 2025	
pcDNA3.1_TMEM9 (T9A) expression plasmid	Planells-Cases et al, 2025	
pcDNA3.1_CD4	N/A	
T9AΔpAC expression construct	Planells-Cases et al, 2025	
T9AΔpAC, ΔCID expression construct	Planells-Cases et al, 2025	
CLCN3 variant constructs	This study	
**Antibodies**		
Anti-LAMP2 monoclonal antibody (H4B4)	Abcam	ab25631
Anti-TMEM9 guinea pig antibody	Planells-Cases et al, 2025	T9AC2, gp
anti-GFP chicken antibody	Aves lab	GFP-1020
Goat anti-Mouse IgG (H + L) Highly Cross-Adsorbed Secondary Antibody, Alexa Fluor™ 633	Invitrogen	A-21052
Goat anti-Guinea Pig IgG (H + L) Highly Cross-Adsorbed Secondary Antibody, Alexa Fluor™ 555	Invitrogen	A-21435
Goat anti-Chicken IgY (H + L) Secondary Antibody, Alexa Fluor™ 488	Invitrogen	A-11039
**Oligonucleotides and other sequence-based reagents**		
QuikChange primers	This study	Table EV1
**Chemicals, enzymes and other reagents**		
DMEM	PAN Biotech	P04-03550
FBS Good	PAN Biotech	P40-37500
Penicillin/streptomycin	PAN Biotech	P06-07100
DPBS, no calcium, no magnesium	Gibco	14190144
Trypsin-EDTA (0.05%), phenol red	Gibco	25300054
HBSS	Gibco	14025092
Opti-MEM™ I Reduced Serum Medium, GlutaMAX™ Supplement	Gibco	51985034
Lipofectamine 2000	Thermo Fisher	11668019
Poly-ʟ-Lysine Hydrobromide	Sigma-Aldrich	P4707
Lysosensor Yellow/Blue DND-160	Invitrogen	L7545
4’,6-Diamidino-2-phénylindole dihydrochloride	Sigma-Aldrich	MBD0015
Bovine Serum Albumin (BSA) Fraction V, NZ-Origin	Carl Roth	8076.3
Saponin from quillaja bark	Sigma-Aldrich	S7900
Goat Serum	PAN Biotech	P30-1002
Fluoromount-G®	SouthernBiotech	0100-01
**Software**		
PATCHMASTER V2X90.3	HEKA Elektronik	
Python 3.8	Python Software Foundation	
SciPy 1.5.2	Python Software Foundation/SciPy project	
**Other**		
LSM880	Zeiss	
EPC-10 USB	HEKA Elektronik	89-5331

### Study cohort

Subjects were identified through GeneMatcher (Sobreira et al, 2015), ERN-ITHACA—a European Rare Disease Network for rare congenital malformations and intellectual disability, ERN EpiCARE—a European rare-disease network of Epilepsy and Genetics departments, or by being contacted as the corresponding author of a previous publication (Duncan et al, 2021). We also contacted authors of previous publications and asked for updates on published subjects. Clinical and genetic data were collected using standardized forms completed by clinicians or caregivers. Collected variables included developmental milestones, cognition and behavior, seizure and movement disorder history (including age of onset and offset and seizure semiology), developmental trajectory, physical examination findings, and results of electroencephalography and neuroimaging. The study was approved by the local ethics committees. The study complies with the principles laid out in the Declaration of Helsinki. As all probands were minors or had cognitive impairment, their parents or legal guardians provided written informed consent.

### Variant analysis

*CLCN3* variants were annotated using the NM_001829.4 (GRCh38) transcript of *CLCN3*. Numbering of amino acids corresponds to the long ClC-3b isoform. We predicted deleterious effects of variants using SIFT (Ng and Henikoff, 2003), PolyPhen-2 (Adzhubei et al, 2010), AlphaMissense (Cheng et al, 2023), REVEL (Ioannidis et al, 2016), CADD (Rentzsch et al, 2019), and SpliceAI (Jaganathan et al, 2019). We employed SpliceAI (https://spliceailookup.broadinstitute.org/#) and dbscSNV (Jian et al, 2014) to predict the effect of the splice-site variant. Genome Aggregation Database (Karczewski et al, 2020) was employed to check the presence of the variants in control populations. Variants were described according to HGVS-nomenclature recommendations using Mutalyzer software (https://mutalyzer.nl/) and classified according to American College of Medical Genetics and Genomics (ACMG) and the Association for Molecular Pathology guidelines (Richards et al, 2015).

### Expression constructs and cell culture

Mouse ClC-3 splice variant c and human ClC-3 splice variant a cDNAs were inserted into a modified pEGFP-C1 (Clontech) vector, where EGFP was replaced by mVenus. Untagged T9A cDNA was cloned into pCDNA3.1 (Thermo Fisher) (Planells-Cases et al, 2025). Untagged CD4 was used as a transfection control. Missense variants were introduced using the QuikChange approach (Agilent), mutagenesis primers are listed in Table EV1. All clones were verified by Sanger sequencing the entire ORF.

WT HeLa and *TMEM206*^−/−^ HEK293 cells (Ullrich et al, 2019) were maintained in DMEM with 10% FBS and 1% penicillin/streptomycin at 37 °C and 5% CO_2_. Cells were transfected 48 h before experiments using Lipofectamine 2000 (Thermo Fisher) according to the manufacturer’s instructions.

### Immunocytochemistry

Immunofluorescent staining was performed as described in (Planells-Cases et al, 2025). WT HeLa cells were seeded on uncoated glass coverslips 2 h prior to transfection and co-transfected with Venus-ClC-3a (WT or mutants) plus untagged T9A, or CD4 as a control, at 1:1 DNA ratio. 48 h post transfection, cells were fixed with methanol in phosphate-buffered saline (PBS) for 10 min, permeabilized with 0.1% saponin and blocked in 3% goat serum, 2% BSA in PBS and incubated overnight at 4 °C with primary antibodies (listed in Reagents and Tools Table) in 0.05% saponin, 3% BSA in PBS. Subsequently, cells were incubated for 1–2 h at room temperature with secondary antibodies coupled to fluorophores (listed in Reagents and Tools Table) and DAPI, washed thrice with PBS, and mounted on slides with Fluoromount-G™. Slides were imaged with the LSM880 confocal microscope with a ×63, 1.4 numerical aperture oil-immersion lens (Zeiss). The experiment was performed in a non-blinded manner, at least in three transfections, with at least ten images taken per slide.

### Lysosensor staining

WT HeLa cells were seeded onto glass-bottom MatTek imaging dishes 2 h prior to transfection and co-transfected with Venus-ClC-3a (WT or mutants) plus untagged T9A, or CD4 as a control, at 1:1 DNA ratio. After 48 h, cells were incubated for 15 min at 37 °C with 5 μM Lysosensor®Yellow/Blue DND-160 (Invitrogen, #L7545) in complete DMEM medium, washed twice with prewarmed PBS and immediately imaged in HBSS medium for the following 15 min. Cells were imaged at the LSM880 confocal microscope (Zeiss) at ×63 magnification using *λ*_ex_  =  405 nm and *λ*_em_  =  511–588 nm for Lysosensor®, and *λ*_ex_  =  488 nm for Venus. Experiments were performed in a non-blinded manner at least in three independent transfections with 4−9 images taken from each dish.

Vesicle area was quantified from the Lysosensor® (blue) channel using a custom Python (Python Software Foundation) script. Putative vesicle locations were identified using a multi‑scale Laplacian‑of‑Gaussian (LoG) blob detector applied to a lightly contrast‑enhanced image (contrast‑limited adaptive histogram equalization, CLAHE) followed by mild Gaussian smoothing to reduce pixel noise. Candidate vesicle centers were defined as local maxima in the LoG response across spatial scales, enabling detection of vesicles across a range of sizes.

**Figure EV3:**
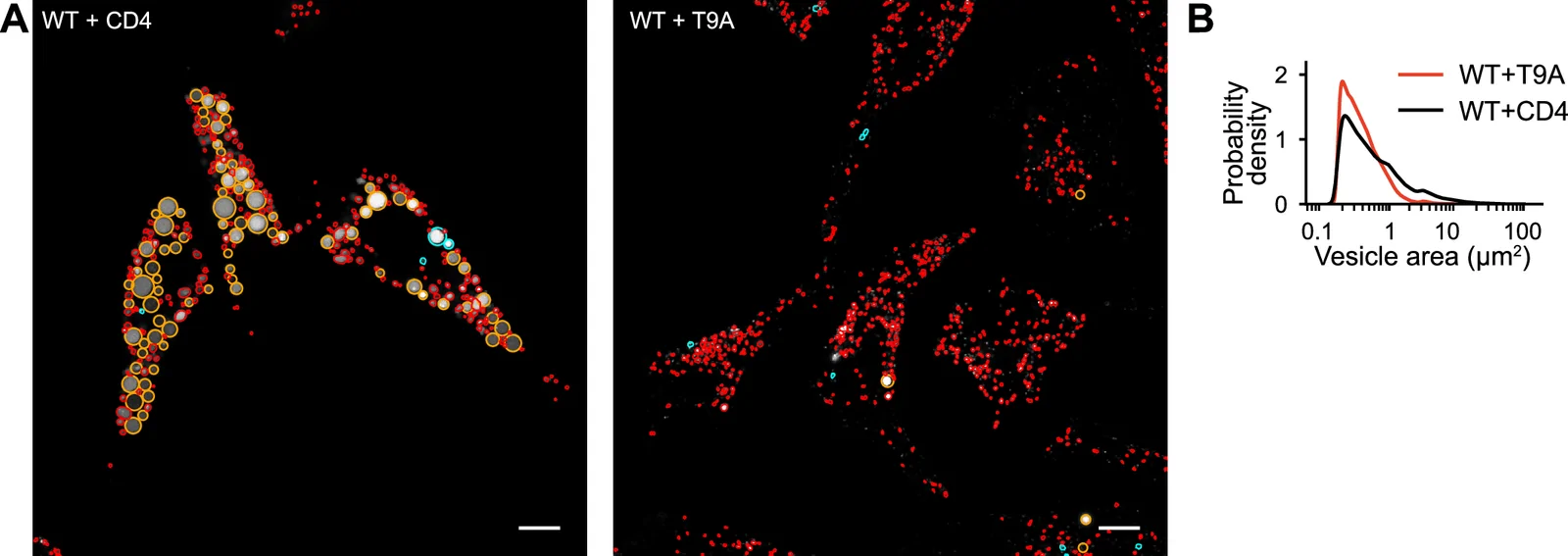
Quantification of cell vacuolization upon ClC-3 overexpression. (**A**) WT HeLa cells overexpressing WT ClC-3 with CD4 control or T9A stained with Lysosensor® Yellow/Blue and imaged at pH-insensitive wavelength. Large vesicles, for which a circular fit was performed, are outlined in orange, whereas the rest of the vesicles detected by the algorithm are shown in red. Detections with top 1% relative standard deviation of intensity are highlighted in cyan and were excluded from analysis. (**B**) Estimated area of Lysosensor-positive vesicles in HeLa cells overexpressing WT ClC-3 with T9A (red) or CD4 (black) as evaluated by our script (see “Methods”). Size distribution was obtained by pooling all lysosomes from >5 fields of view from 13 independent transfections.

To obtain the total vesicle area around a candidate center, a small image patch around the center was extracted and threshold‑based segmentation was performed on a Gaussian‑smoothed version of the raw (non-contrast‑enhanced) image. The threshold was selected by combining Otsu thresholding with constraints based on the center intensity and local background, so that the candidate center remained within the segmented foreground. When multiple contiguous regions were present within the patch, only the region containing the center was retained as the vesicle lumen. In crowded regions where a single segmented region could include more than one vesicle connected with a narrow neck, touching objects could be separated using a distance‑transform watershed approach. Very large, approximately round lumen‑positive structures were summarized by fitting a circle to outline points. Duplicate detections were suppressed using non‑maximum suppression based on center proximity and overlap (intersection‑over‑union criteria), and small detections fully contained within larger vesicles were removed. To improve performance in bright and densely populated regions, detection was run in two passes by masking objects above a user‑defined size cutoff after the first pass with masked pixels replaced by a local background estimate to avoid introducing sharp edges, followed by pooling detections and final de‑duplication. For each detected object, relative standard deviation of the intensity was calculated and top 1% of detection were flagged for each image as possible false-positives. For each detected object, area, area‑equivalent radius and intensity summary statistics were exported, and overlays of detected objects were visualized for quality control (Fig. EV3).

Vesicle area data are presented as probability density distributions, with control measurements taken from matched WT samples imaged in the same experimental sessions as the corresponding mutant. Because the analysis workflow does not include cell segmentation or gating by transfection status, vesicles from both transfected and non-transfected cells contribute to each distribution; this is expected to dilute variant-associated shifts and may therefore underestimate the magnitude of variants’ effects.

### Electrophysiology

For patch-clamp recordings, cells transfected 48 h earlier were seeded on poly-L-lysine-coated (Sigma-Aldrich, P4707) coverslips 1–5 h before measurement. Whole-cell patch-clamp experiments were performed using an EPC-10 USB patch-clamp amplifier and PATCHMASTER software (HEKA Elektronik). Currents were sampled at 20 kHz and low-pass filtered at 2 kHz. Patch pipettes had a resistance of 3–5 MΩ. The series resistance did not exceed 10 MΩ and was compensated by at least 30%. Voltage step protocol consisted of 100-ms test voltage steps at −120 mV to +160 mV in 20 mV increments from the holding potential of −30 mV. Voltage steps were followed by a 100-ms step at −80 mV and applied at 2 s intervals. Currents were normalized to cell capacitance to yield current density.

The patch pipette solution contained in mM: 140 CsCl, 5 EGTA, 1 MgCl_2_, 10 HEPES, with pH adjusted to 7.2 with CsOH (275 mOsm/kg). Standard bath solution contained (in mM): 150 NaCl, 6 CsCl, 1 MgCl_2_, 1.5 CaCl_2_, 10 D-glucose, 10 HEPES (pH adjusted to 7.5 using NaOH) (320 mOsm/kg). For bath pH 4.5, HEPES was substituted with 5 mM citrate/citric acid buffer.

Data was analyzed using the SciPy 1.5.2 library for Python 3.8 programming language (Python Software Foundation).

## Supplementary information

Table EV1

Peer Review File

Dataset EV1

Dataset EV2

Source data Fig. 2

Source data Fig. 3

Source data Fig. 4

Expanded View Figures

## Data Availability

This study includes no data deposited in external repositories. The source data of this paper are collected in the following database record: biostudies:S-SCDT-10_1038-S44321-026-00486-6.
