# Supplementary material for: Abnormal ClC-3/TMEM9-mediated endosomal ion transport in CLCN3-associated neurodevelopmental disease
Source: EMBO Mol Med. 2026 Jul 22;18(9):3474–94. doi: 10.1038/s44321-026-00486-6 (PMC13562645; doi:10.1038/s44321-026-00486-6)
Supplement: Supplementary file 1 [file 44321_2026_486_MOESM2_ESM.pdf]

EMBO Molecular Medicine

Abnormal ClC-3/TMEM9-mediated endosomal ion transport in CLCN3-associated neurodevelopmental disease

Maya Polovitskaya, Tinatin Tkemaladze, Lotte Jensen, Sumanta Kar, Rosa Planells-Cases, Emanuele Agolini, Pankaj Agrawal, Paolo Alfieri, Tahsin Stefan Barakat, MIriam Bertrand, Pamela Bowman, Alice Brooks, Ange-Line Bruel, Sara Cabet, Karolina Chwialkowska, Giovanna Colafati, Julian Delanne, Laurence Faivre, Cristina Felton, Ute Grasshoff, Anne-Marie Guerrot, Tobias Haack, Claudia Haase, Ashley Helseth, Katrien Janssens, Amjad Khan, Margarete Koch-Hogrebe, Joshua Mandelberg, Elisabeth Mangold, Elene Melikidze, Marije Meuwissen, Ieva Mičule, Brittany Noble, Konrad Platzer, Dmitrijs Rots, Catherin Sarret, Ariane Schmetz, Amelle Shillington, Frederic Tran Mau-Them, Dagmar Wieczorek, Gaetan Lesca, Allan Bayat, and Thomas Jentsch

Corresponding authors: Thomas Jentsch (jentsch@fmp-berlin.de) , Allan Bayat (abaya@filadelfia.dk)

Review Timeline:

Submission Date:	25th Apr 26
Editorial Decision:	19th May 26
Revision Received:	17th Jun 26
Editorial Decision:	26th Jun 26
Revision Received:	1st Jul 26
Accepted:	6th Jul 26

Editor: Jingyi Hou

Transaction Report:

(Note: With the exception of the correction of typographical or spelling errors that could be a source of ambiguity, letters and reports are not edited. Depending on transfer agreements, referee reports obtained elsewhere may or may not be included in this compilation. Referee reports are anonymous unless the Referee chooses to sign their reports.)

19th May 2026

Dear Prof. Jentsch,

Thank you again for submitting your work to EMBO Molecular Medicine. We have now received feedback from the two referees who agreed to evaluate your manuscript. As you will see from the reports below, the referees found the study interesting and relevant and expressed strong support for it. They also raised a number of relatively minor issues, which we ask you to address in your revision.

I think the referees' recommendations are clear and need not be repeated here. All of the issues raised need to be carefully addressed. As you may already know, our editorial policy allows in principle only one round of major revision, so it is essential that your response fully addresses the referees' comments. Please feel free to contact me if you would like to discuss any of the issues raised by the referees in further detail.

EMBO Molecular Medicine has a "scooping protection" policy, whereby similar findings that are published by others during review or revision are not a criterion for rejection. Should you decide to submit a revised version, I do ask that you get in touch after three months if you have not completed it, to update us on the status.

Please also contact us as soon as possible if similar work is published elsewhere. If other work is published, we may not be able to extend the revision period beyond three months.

Please read below for important editorial formatting and consult our author's guidelines for proper formatting of your revised article for EMBO Molecular Medicine.

I look forward to receiving your revised manuscript.

Sincerely,
Jingyi

Jingyi Hou
Senior Editor
EMBO Molecular Medicine

*****
When preparing your revised manuscript, please refer to our guidelines: https://link.springer.com/journal/44321/submission-guidelines#cms-Revised-submissions. We perform an initial quality control of all revised manuscripts before re-review; failure to include requested items will delay the evaluation of your revision.

We require:

1) A .docx formatted version of the manuscript text (including legends for main figures, EV figures and tables). Please make sure that the changes are highlighted to be clearly visible.

2) Individual production quality figure files as .eps, .tif, .jpg (one file per figure). For guidance, download the 'Figure Guide PDF': https://media.springernature.com/original/springer-cms/rest/v1/content/27825798/data/v1.

3) A .docx formatted letter INCLUDING the reviewers' reports and your detailed point-by-point responses to their comments. As part of the EMBO Press transparent editorial process, the point-by-point response is part of the Review Process File (RPF), which will be published alongside your paper.

4) A complete author checklist, which you can download from our author guidelines. Please insert information in the checklist that is also reflected in the manuscript. The completed author checklist will also be part of the RPF.

5) Please note that all corresponding authors are required to supply an ORCID ID for their name upon submission of a revised manuscript.

6) It is mandatory to include a 'Data Availability' section after the Materials and Methods. Before submitting your revision, primary

datasets produced in this study need to be deposited in an appropriate public database, and the accession numbers and database listed under 'Data Availability'. Please remember to provide a reviewer password if the datasets are not yet public.

In case you have no data that requires deposition in a public database, please state so in this section. Note that the Data Availability Section is restricted to new primary data that are part of this study.

7) For data quantification: please specify the name of the statistical test used to generate error bars and P values, the number (n) of independent experiments (specify technical or biological replicates) underlying each data point and the test used to calculate p-values in each figure legend. The figure legends should contain a basic description of n, P and the test applied. Graphs must include a description of the bars and the error bars (s.d., s.e.m.).

8) At EMBO Press we ask authors to provide source data for the main manuscript figures. You will receive a separate email with instructions for providing source data with your revised manuscript, including how to upload and organize the files.

9) Our journal encourages inclusion of *data citations in the reference list* to directly cite datasets that were re-used and obtained from public databases. Data citations in the article text are distinct from normal bibliographical citations and should directly link to the database records from which the data can be accessed. In the main text, data citations are formatted as follows: "Data ref: Smith et al, 2001" or "Data ref: NCBI Sequence Read Archive PRJNA342805, 2017". In the Reference list, data citations must be labeled with "[DATASET]". A data reference must provide the database name, accession number/identifiers and a resolvable link to the landing page from which the data can be accessed at the end of the reference.

10) We replaced Supplementary Information with Expanded View (EV) Figures and Tables that are collapsible/expandable online. A maximum of 5 EV Figures can be typeset. EV Figures should be cited as 'Figure EV1, Figure EV2" etc... in the text and their respective legends should be included in the main text after the legends of regular figures.

- For the figures that you do NOT wish to display as Expanded View figures, they should be bundled together with their legends in a single PDF file called *Appendix*, which should start with a short Table of Content. Appendix figures should be referred to in the main text as: "Appendix Figure S1, Appendix Figure S2" etc.

- Additional Tables/Datasets should be labeled and referred to as Table EV1, Dataset EV1, etc. Legends have to be provided in a separate tab in case of .xls files. Alternatively, the legend can be supplied as a separate text file (README) and zipped together with the Table/Dataset file.

11) The paper explained: EMBO Molecular Medicine articles are accompanied by a summary of the articles to emphasize the major findings in the paper and their medical implications for the non-specialist reader. Please provide a draft summary of your article highlighting

- the medical issue you are addressing,

- the results obtained and

- their clinical impact.

This may be edited to ensure that readers understand the significance and context of the research. Please refer to any of our published articles for an example.

12) Author contributions: You will be asked to provide CRediT (Contributor Role Taxonomy) terms in the submission system. These replace a narrative author contribution section in the manuscript.

13) A Conflict of Interest statement should be provided in the main text.

14) Every published paper includes a 'Synopsis' to further enhance discoverability. Synopses are displayed on the journal webpage and are freely accessible to all readers. They include a short stand first (maximum of 300 characters, including space) as well as 2-5 one-sentences bullet points that summarizes the paper. Please write the bullet points to summarize the key NEW findings. They should be designed to be complementary to the abstract - i.e. not repeat the same text. We encourage inclusion of key acronyms and quantitative information (maximum of 30 words / bullet point). Please use the passive voice. Please attach these in a separate file or send them by email, we will incorporate them accordingly.

Please also provide a visual abstract to illustrate your article as a PNG file 550 px wide x 300 px high.

15) All Materials and Methods need to be described in the main text using our 'Structured Methods' format. According to this format, the Methods section includes a Reagents and Tools Table (listing key reagents, experimental models, software and relevant equipment and including their sources and relevant identifiers) followed by a Methods and Protocols section describing

the methods, ideally using a step-by-step protocol format. The aim is to facilitate adoption of the methodologies across labs.

Please download and fill our Reagents and Tools Table template (.docx), which you can find in our author guidelines: https://link.springer.com/journal/44320/submission-guidelines#structuredmethods.

When submitting your revised manuscript, please do not include the Reagents and Tools Table in the Methods section of the manuscript but upload it as a separate file choosing the file type "Reagent Table".

***** Reviewer's comments *****

Referee #1 (Remarks for Author):

This is a strong and interesting manuscript that expands the genetic and clinical spectrum of CLCN3-associated neurodevelopmental disease and provides a mechanistic re-evaluation of disease-associated ClC-3 variants in the context of the recently identified TMEM9/TMEM9B inhibitory β-subunits. The combination of clinical genetics, electrophysiology, imaging-based vacuolization assays, and structural interpretation provides a coherent framework for distinguishing likely loss-of-function, gain-of-function, and functionally unresolved variants. Overall, I find the manuscript suitable for publication after minor revision.
Moderate comments
1. The use of ClC-3c for plasma membrane electrophysiology and ClC-3a for vacuolization assays is technically well justified. However, the manuscript should more explicitly acknowledge that these two assays examine different splice variants in distinct membrane contexts. Plasma membrane current properties measured with ClC-3c should therefore be interpreted as complementary to, rather than quantitatively equivalent to, ClC-3a activity in endosomal compartments.
2. LINE367: The statement that bath pH 4.5 "mimics the acidity of endolysosomes" should be phrased more carefully. The electrophysiological assay uses ClC-3c, which is described as localizing to recycling endosomes and partially to the plasma membrane upon overexpression, whereas pH 4.5 more closely approximates late endosomal/lysosomal acidity. The authors may consider rephrasing this as "acidic luminal-like conditions" or "late endosomal/lysosomal-like acidity" to avoid implying that pH 4.5 reflects the native environment of ClC-3c.
3. LINE392: The authors refer to the vacuolization assay as "more sensitive." This is plausible, as small residual ClC-3 activity or partial escape from TMEM9-mediated inhibition may accumulate over time and produce a morphological phenotype even when plasma membrane currents remain below the detection threshold. However, the term "more sensitive" should be defined more explicitly. The vacuolization assay is an indirect, cumulative morphological readout and may be influenced by expression level, trafficking, organelle identity, and image-analysis parameters.
4. Related to this point, enlarged LysoSensor-positive compartments could potentially reflect altered endosomal recycling, internalization, trafficking, maturation, fusion/fission, or organelle homeostasis, in addition to altered ClC-3-mediated ion exchange. This is particularly relevant because ClC-3 splice variants localize to distinct endosomal compartments, and TMEM9 proteins can regulate both transport inhibition and trafficking/surface expression. I suggest that the authors discuss this limitation more explicitly and avoid presenting vacuolization as a direct or exclusive readout of ion transport gain-of-function.
5. Please clarify whether the statistical unit in the vacuolization assay is vesicle, cell, image, dish, or independent experiment, and how biological replicates were handled. This is important because vesicles within the same cell or image are not fully independent observations. Please also clarify the family of comparisons used for Benjamini-Hochberg correction.
6. Have the authors examined the time course or reversibility of vacuole formation, for example by chase/recovery experiments? Such data are not essential, but clarification would help distinguish whether the assay detects an acute increase in ClC-3 activity or a cumulative defect in endosomal/lysosomal homeostasis.

Minor points:
1. LINE139: Please define the abbreviations T9A and T9B more explicitly at first use and ensure consistent terminology throughout the manuscript. The manuscript alternates between TMEM9, T9A, T9 proteins, T9 subunits, and T9-mediated inhibition.
2. LINE193: Although all constructs were verified by Sanger sequencing of the entire ORF, providing the QuikChange mutagenesis primer sequences, or at least a full list of generated and sequence-verified constructs, in the Supplementary Information would further improve reproducibility.

Referee #2 (Comments on Novelty/Model System for Author):

The view on ClC-3 function and the consequences of mutations was dramatically altered by the recent discovery of the TMEM9 inhibitory beta-subunit. This created a significant knowledge gap concerning the interpretation of these mutations. This gap is filled here and additional mutations are described.

Referee #2 (Remarks for Author):

Polovitskaya et al. report 15 individuals with a heterogeneous neuropsychiatric disorder caused by 13 missense, 1 in-frame

deletion, and 1 splice variant. Since the inhibitory subunit TMEM9 had not been known at the time of the first description of the CLCN3-related syndrome, all 20 known variants not leading to obvious LOF were tested electrophysiologically and by a endolysosomal swelling assay upon heterologous expression in HEK293 cells. 12 of the variants showed impaired inhibition by TMEM9 and of these 8 also displayed an abnormal inward current at acidic pH. The remaining variants did not produce conclusive effects. The clinical manifestations of the 16 individuals carrying function-altering variants are discussed in detail.

General

This manuscript fills an important gap in our understanding of the CLCN3-associated neurodevelopmental disorder. It underlines the crucial role of TMEM9 in the pathogenesis and confirms the relevance of amino acid residues in ClC-3 shown to mediate the interaction with TMEM9 by previous structural analysis. The paper contains high quality phenotype and functional data, clear figures, and is well written. It will be of interest for ion channel researchers as well as for clinicians.

Major

In the abstract and throughout the text the authors forget to mention the Asp87del variant that leads to a GOF. An amino acid deletion is no missense variant.

The clinical presentation is given in great detail in Table 1 and described in paragraph 3.2. To grasp the phenotype more easily I would suggest to present the ten most frequent hallmarks of the disorder with their respective frequencies. E.g. hypotonia 8/13=63%, which is more precise than "Ten subjects exhibited abnormal findings in the neonatal period. Muscular hypotonia was the most frequent feature.". This information can be added to the table or be presented as a separate figure.

No information is given on the subcellular localisation and expression levels of the ClC-3 mutants. In Figure EV2 the Y386H mutant protein shows a quite strong Golgi localisation if co-expressed with TMEM9. I would strongly suggest to address this point in the manuscript because this could be an indication for the pathomechanism. If reproducible changes have been seen this could also be mentioned in Tables S1 and S2.

The ACMG criterium PM2 has been generally downgraded to PM2_supporting.

Minor

Dystonia was not only seen in #12 and #14, but also in #3 with the unclear homozygous p.Trp385Cys variant.

It is worth mentioning that all 4 individuals showing regression have autism of which the regression is probably a consequence.

In Table S1 the ACMG criteria PM2, PM3, PP2, PP3, PP4 are claimed for c.1155G>T p.Trp385Cys, which corresponds to 7 points and thus to likely pathogenic. However, PM3 cannot be chosen because the variant is homozygous and not in combination with a bone fide pathogenic heterozygous variant. PP4 is precluded since the phenotype is an unspecific retardation with autism that can be caused by dozens if not hundreds of disease genes. PM2 can only be counted as PM2_sup. The final verdict VUS remains the same.
While the amino acid change induced by c.1155G>T has highly pathogenic predictions c.1156T>C seems rather benign on protein level. For both variants SpliceAI predicts a mild splice defect (acceptor loss) that does not reach the -0.2 threshold. The phenotype of individual 3 harboring c.1155G>T is surprisingly mild and both, c.1155G>T and c.1156G>T, are not associated with seizures.

The statement "However, these variants are extremely rare in population databases..." is not precise since only one of the eight variants without clear functional effects is found in gnomAD. This variant c.1238C>T p.Ala413Val is a conservative exchange and shows weak bioinformatic scores. All others are absent and in those for which parents were available for analysis de novo.

Detailed response to the reviewers of EMM-2026-23864 (Polovitskaya et al.)

We thank both reviewers for their positive comments. Please find our answers and details of changes made in response to their constructive suggestions below.

Referee #1 (Remarks for Author):

This is a strong and interesting manuscript that expands the genetic and clinical spectrum of CLCN3-associated neurodevelopmental disease and provides a mechanistic re-evaluation of disease-associated ClC-3 variants in the context of the recently identified TMEM9/TMEM9B inhibitory β-subunits. The combination of clinical genetics, electrophysiology, imaging-based vacuolization assays, and structural interpretation provides a coherent framework for distinguishing likely loss-of-function, gain-of-function, and functionally unresolved variants. Overall, I find the manuscript suitable for publication after minor revision.

We thank the reviewer for these positive comments.

Moderate comments

1. The use of ClC-3c for plasma membrane electrophysiology and ClC-3a for vacuolization assays is technically well justified. However, the manuscript should more explicitly acknowledge that these two assays examine different splice variants in distinct membrane contexts. Plasma membrane current properties measured with ClC-3c should therefore be interpreted as complementary to, rather than quantitatively equivalent to, ClC-3a activity in endosomal compartments.

We agree that it is important to stress these differences more than previously done.
We now added in Results, line 277-279, the following sentence:
'These splice variants have identical ion transport properties when directed to the plasma membrane (Guzman et al, 2015). However, their localization to different membranes, which differ in lipid and phosphatidylinositide composition, may differentially affect their activity.'
We also addressed this point later in discussion, see response to point 3 that discusses the apparently higher sensitivity of the vacuolization assay.

2. LINE367: The statement that bath pH 4.5 "mimics the acidity of endolysosomes" should be phrased more carefully. The electrophysiological assay uses ClC-3c, which is described as localizing to recycling endosomes and partially to the plasma membrane upon overexpression, whereas pH 4.5 more closely approximates late endosomal/lysosomal acidity. The authors may consider rephrasing this as "acidic luminal-like conditions" or "late endosomal/lysosomal-like acidity" to avoid implying that pH 4.5 reflects the native environment of ClC-3c.

We agree with the reviewer that the pH value (4.5) used in these experiments to reveal the pH sensitivity of ClC-3 variants is probably somewhat more acidic that the pH of late endososomes where most ClC-3/T9 is thought to reside. As suggested by the reviewer, we reworded the sentence (now line 286) to: 'Switching bath pH to 4.5, close to late endosomal/lysosomal-like acidity, moderately….'

3. LINE392: The authors refer to the vacuolization assay as "more sensitive." This is plausible, as small residual ClC-3 activity or partial escape from TMEM9-mediated inhibition may accumulate over time and

produce a morphological phenotype even when plasma membrane currents remain below the detection threshold. However, the term "more sensitive" should be defined more explicitly. The vacuolization assay is an indirect, cumulative morphological readout and may be influenced by expression level, trafficking, organelle identity, and image-analysis parameters.

Thank you for the excellent suggestion to highlight the cumulative nature of vesicle 'swelling' and the importance of subcellular localization. We changed the sentence to (line 395-398):
'The apparently higher sensitivity of the vacuolization assay may be owed to the integration of small increases in volume over time, or to differences in the membrane environment. Both types of assays only qualitatively reflect ion transport as the human variants may also affect subcellular trafficking of the transporters.'

4. Related to this point, enlarged LysoSensor-positive compartments could potentially reflect altered endosomal recycling, internalization, trafficking, maturation, fusion/fission, or organelle homeostasis, in addition to altered ClC-3-mediated ion exchange. This is particularly relevant because ClC-3 splice variants localize to distinct endosomal compartments, and TMEM9 proteins can regulate both transport inhibition and trafficking/surface expression. I suggest that the authors discuss this limitation more explicitly and avoid presenting vacuolization as a direct or exclusive readout of ion transport gain-of-function.

Good point, we addressed this issue as stated above for point 3.

5. Please clarify whether the statistical unit in the vacuolization assay is vesicle, cell, image, dish, or independent experiment, and how biological replicates were handled. This is important because vesicles within the same cell or image are not fully independent observations. Please also clarify the family of comparisons used for Benjamini-Hochberg correction.

No quantitative comparison was performed for the vacuolization assay. Vesicle area distributions were built by pooling all detected lysosomes from date-matched experiments to represent the complete dataset and avoid choosing 'representative' images. Each variant was assessed in at least three independent transfections with 4–9 images taken from each dish, each containing multiple cells, not taking into account their transfection status. Thus, these distributions are affected by cells not expressing ClC-3 in the field of view which differed between dates but largely not between conditions.
All legends read: Mann-Whitney U test; false discovery rate was controlled using the Benjamini–Hochberg procedure.

6. Have the authors examined the time course or reversibility of vacuole formation, for example by chase/recovery experiments? Such data are not essential, but clarification would help distinguish whether the assay detects an acute increase in ClC-3 activity or a cumulative defect in endosomal/lysosomal homeostasis.

We have only roughly investigated the time course of vacuole formation, which becomes apparent, depending on transfection efficiency and mutant, about one day after transfection, and increases over time. The shown images were obtained two days after transfection. Of course, the process is non-linear and will come to an end when the cells are filled with vacuoles or die. We did not observe reversibility, within this time period, but you could expect it after longer time intervals with transient, low-level expression and variants causing only moderate enlargement (thereby avoiding cell death). In our study on a CLCN6 variant (https://pubmed.ncbi.nlm.nih.gov/33217309/) we followed the increase in vesicle size over time in live cell imaging using a U2-OS cells carrying lamp2-GFP as KI, and in some experiment added

the proton pump inhibitor bafilomycin A which reversed the swelling since net CLC transport is fueled by the H^+ gradient generated by the H^+-ATPase. The amount and time course of swelling depends on transfection efficiency, which is difficult to control. Quantifying the kinetics of vacuolization is difficult and we would consider it to be pseudo-exact. The assay is very nice, but only semiquantitative.

Minor points:

1. LINE139: Please define the abbreviations T9A and T9B more explicitly at first use and ensure consistent terminology throughout the manuscript. The manuscript alternates between TMEM9, T9A, T9 proteins, T9 subunits, and T9-mediated inhibition.

We have defined T9A and T9B in line 142, and in line 146 T9 as generic T9A and T9B. We agree that it may have been confusing to switch between spelling out the TEMEM9 and T9 terminology and have now – with the exception of the title and abstract – always use the short abbreviations (which we had also used in our first paper on the subunits (Planells-Cases et al, Nature Comm 2025).

2. LINE193: Although all constructs were verified by Sanger sequencing of the entire ORF, providing the QuikChange mutagenesis primer sequences, or at least a full list of generated and sequence-verified constructs, in the Supplementary Information would further improve reproducibility.

The sequences of all these primers have now been added to the Table S3.

Referee #2 (Comments on Novelty/Model System for Author):

The view on ClC-3 function and the consequences of mutations was dramatically altered by the recent discovery of the TMEM9 inhibitory beta-subunit. This created a significant knowledge gap concerning the interpretation of these mutations. This gap is filled here and additional mutations are described.

Referee #2 (Remarks for Author):

Polovitskaya et al. report 15 individuals with a heterogeneous neuropsychiatric disorder caused by 13 missense, 1 in-frame deletion, and 1 splice variant. Since the inhibitory subunit TMEM9 had not been known at the time of the first description of the CLCN3-related syndrome, all 20 known variants not leading to obvious LOF were tested electrophysiologically and by a endolysosomal swelling assay upon heterologous expression in HEK293 cells. 12 of the variants showed impaired inhibition by TMEM9 and of these 8 also displayed an abnormal inward current at acidic pH. The remaining variants did not produce conclusive effects. The clinical manifestations of the 16 individuals carrying function-altering variants are discussed in detail.

General

This manuscript fills an important gap in our understanding of the CLCN3-associated neurodevelopmental disorder. It underlines the crucial role of TMEM9 in the pathogenesis and confirms the relevance of amino acid residues in ClC-3 shown to mediate the interaction with TMEM9 by previous structural analysis. The paper contains high quality phenotype and functional data, clear figures, and is well written. It will be of interest for ion channel researchers as well as for clinicians.

We thank the reviewer for these positive comments.

Major

In the abstract and throughout the text the authors forget to mention the Asp87del variant that leads to a GOF. An amino acid deletion is no missense variant.

Sorry for this mistake, which we have now corrected in line 178 to '1 in-frame deletion of a single amino-acid'.

The clinical presentation is given in great detail in Table 1 and described in paragraph 3.2. To grasp the phenotype more easily I would suggest to present the ten most frequent hallmarks of the disorder with their respective frequencies. E.g. hypotonia 8/13=63%, which is more precise than "Ten subjects exhibited abnormal findings in the neonatal period. Muscular hypotonia was the most frequent feature.". This information can be added to the table or be presented as a separate figure.

We thank the reviewer for this comment. Indeed, the section summarizing the clinical findings did not read well and the reader may have got lost. We have now replaced this entire section with a new, better structured and even somewhat shorter text (lines 207-262), that highlights the most frequent findings in the first paragraph we have pasted here:

'Clinical findings are summarized in Table 1 and Supplemental Table S1. Frequencies are reported as n/N, with N indicating the number of individuals for whom the respective feature was available. Among the 16 individuals carrying function-changing *CLCN3* variants or biallelic likely loss-of-function variants, the most frequent clinical hallmarks were developmental delay and/or intellectual disability (13/13, 100%), gross motor delay or impaired ambulation (13/14, 93%), intellectual disability (11/12, 92%), vision or eye abnormalities (11/13, 85%), language impairment (11/14, 79%), behavioral or psychiatric features (10/14, 71%), gastrointestinal or feeding symptoms (10/16, 63%), hypotonia (8/13, 62%), and autistic features (8/13, 62%).'

We believe that this section is now more informative.

No information is given on the subcellular localisation and expression levels of the ClC-3 mutants. In Figure EV2 the Y386H mutant protein shows a quite strong Golgi localisation if co-expressed with TMEM9. I *would strongly suggest to address this point in the manuscript* because this could be an indication for the pathomechanism. If reproducible changes have been seen this could also be mentioned in Tables S1 and S2.

The reviewer is of course absolutely right in stating that subcellular trafficking may influence the outcome of our assays. We are well aware of this issue as our previous work (Planells-Cases et al, Nature Comm 2025) had revealed that overexpression of ClC-4 and ClC-5 (without T9 proteins) does not generate large vesicles except when they are carried to the swelling-competent endolysosomal compartment by co-expression of ClC-3 and T9, respectively.

*In response to reviewer one (point 3) we have added the sentence: '*Both types of assays only qualitatively reflect ion transport as the human variants may also affect subcellular trafficking of the transporters' *which partially also addresses this point of reviewer 2.*

We would like to stress that in general we did not observe significant changes in the subcellular localization of the transporters as a function of the amino acid change. A major problem is, of course, the over-

expression of the variants in our assays, which may lead to localization in the ER or Golgi and varies from cell to cell.

In order to directly answer the concern of the reviewer concerning the localization of the Y386H mutant, we now directly compared in new experiments (with SK and RPC added as new co-authors) the subcellular localization of the Y386H variant in comparison to the Golgi marker GM130 (Fig. R1 for the reviewer). For comparison, we also included new experiments for S453R, K645E, P666A, and V772A, all of them with T9A co-expression. We failed to detect prominent Golgi localization. Neither did we detect differences between these mutants.

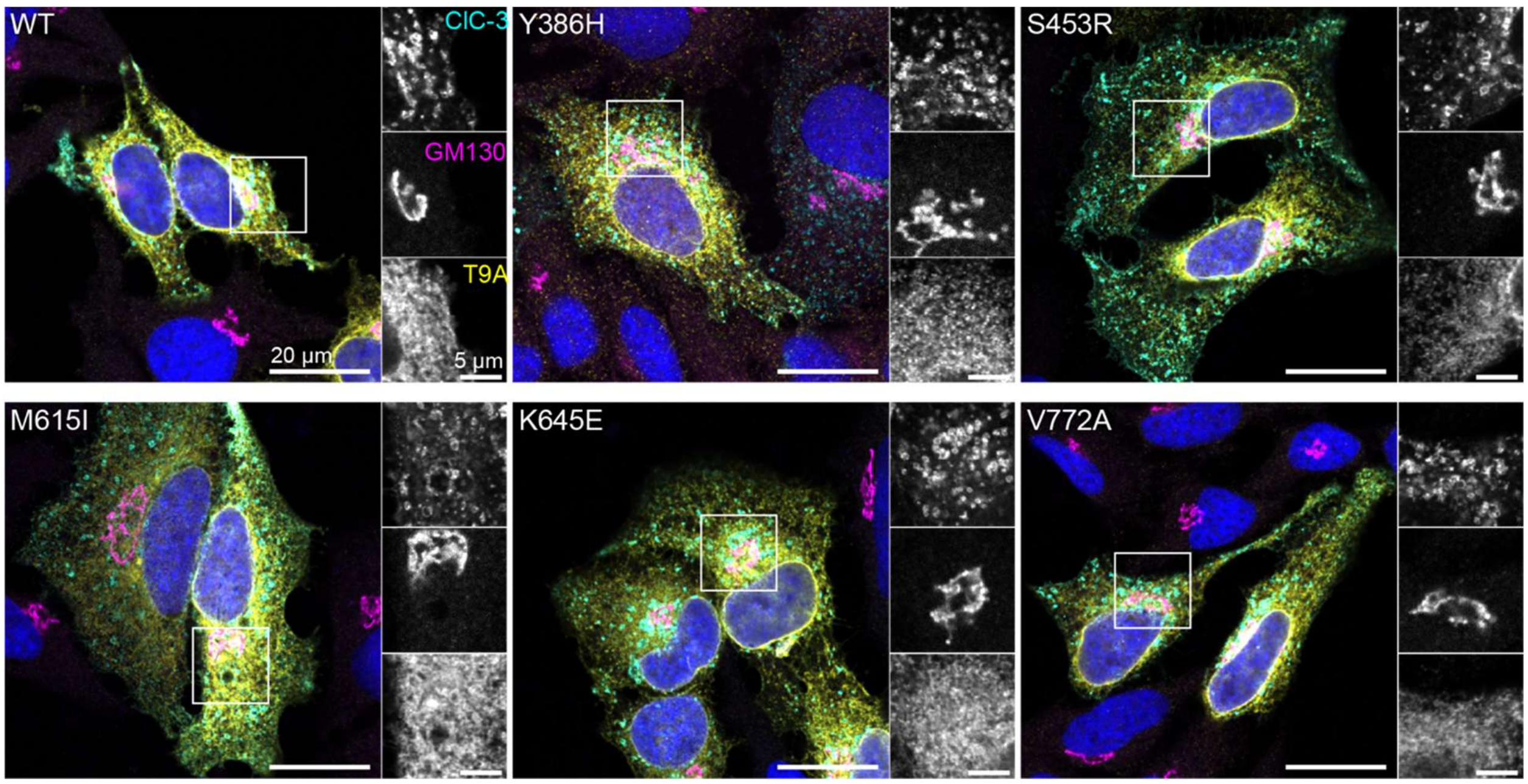

Figure R1 for the reviewer
Immunocytochemistry of HeLa cells two days after transfection of GFP-tagged ClC-3, WT or mutant, together with T9A. Cell were stained for ClC-3, T9A, or the Golgi marker GM130. No marked co-localization of ClC-3 with the Golgi marker. Staining for T9A, which is in large molecular excess, is ER-like. No marked difference between the ClC-3 mutants.

In addition to these new experiments, we also revised the other images of transfected cells we had taken previously. It became clear that the image for the Y388H variant (mentioned by the reviewer) in Figure EV2C was atypical, showing marked expression of the mutant close to the nucleus. We therefore exchanged this panel by a more representative field of view depicting two cells with differing overexpression levels in the revised Fig. EV2.

The ACMG criterium PM2 has been generally downgraded to PM2_supporting.

Thank you, we have corrected the Table.

Minor

Dystonia was not only seen in #12 and #14, but also in #3 with the unclear homozygous p.Trp385Cys variant.

Thank you, we have corrected this mistake in the revised section on clinical finding (line 225).

It is worth mentioning that all 4 individuals showing regression have autism of which the regression is probably a consequence.

Thank you for this interesting observation, which we have added in the section describing the clinical findings (line 241).

In Table S1 the ACMG criteria PM2, PM3, PP2, PP3, PP4 are claimed for c.1155G>T p.Trp385Cys, which corresponds to 7 points and thus to likely pathogenic. However, PM3 cannot be chosen because the variant is homozygous and not in combination with a bone fide pathogenic heterozygous variant. PP4 is precluded since the phenotype is an unspecific retardation with autism that can be caused by dozens if not hundreds of disease genes. PM2 can only be counted as PM2_sup. The final verdict VUS remains the same.

Thank you so much for carefully evaluating our table, which we have corrected.

While the amino acid change induced by c.1155G>T has highly pathogenic predictions c.1156T>C seems rather benign on protein level. For both variants SpliceAI predicts a mild splice defect (acceptor loss) that does not reach the -0.2 threshold. The phenotype of individual 3 harboring c.1155G>T is surprisingly mild and both, c.1155G>T and c.1156G>T, are not associated with seizures.

Both variants are located quite far from the nearest exon boundary and are unlikely to affect splicing. A tyrosine to histidine substitution retaining an aromatic side chain is potentially better tolerated than an introduction of a highly reactive cysteine residue. Mild phenotype of the W385C is consistent with no obvious gain-of-function and potentially partial loss of function without the loss of protein. No change made in the manuscript.

The statement "However, these variants are extremely rare in population databases..." is not precise since only one of the eight variants without clear functional effects is found in gnomAD. This variant c.1238C>T p.Ala413Val is a conservative exchange and shows weak bioinformatic scores. All others are absent and in those for which parents were available for analysis de novo.

*We have changed the wording to: '*these variants are extremely rare in or absent from population databases*'. (line 424) p.Pro666Ala occurs once in AllofUs and p.Met804Thr appears once in gnomAD.*

26th Jun 2026

Dear Prof. Jentsch,

Thank you for the submission of your revised manuscript to EMBO Molecular Medicine. We are satisifed with the revisions made and I am pleased to inform you that we will be able to accept your manuscript pending the following editorial-level amendments:

1. Please remove "Authors' contribution" from the manuscript file.

2. Please ensure that the EV figures are called out in sequential order; currently Fig EV2A is called out before Fig EV1E, Figure EV3 before Fig EV1C,D.

3. "Competing Interests " should be renamed to "DISCLOSURE AND COMPETING INTERESTS STATEMENT". Karolina Chwialkowska works for IMAGENE ME; this should be mentioned in the statement.

4. Please move the information currently included in the Ethical Declaration section to the appropriate locations within the Methods section. The separate Ethical Declaration section should then be removed.

5. Data availability section: since this study does not generate large-scale datasets, please only include the following sentence in this section- "This study includes no data deposited in external repositories".

6. We have recently updated the requirements for visual abstracts. If possible, please adjust the image size to exactly 550 pixels wide × 300 pixels high for optimal display on our website. We apologize for any inconvenience this may cause.

7. Rename Appendix Tables S1-S3 to Dataset EV1-EV3, and ensure that all corresponding callouts in the manuscript text are updated accordingly. Please also add a legend with a brief description for each dataset on a separate worksheet.

8. Please add "the paper explained" to the manuscript file.

9. Funding: in the submission system, please move the information in the comments section to the line-by-line list of funders above.

10. Please address the following issues in figure legends:
- Please note that the legends for figure EV1 is not provided in the sequential manner (legend for figure EV1C is provided before legend of figure EV1B). This needs to be rectified.
- Please note that the legend for figure panel 3B is mislabeled as 3C; and the legend for the figure panel 3C is mislabeled as 3B in the manuscript. This needs to be rectified.
- Please note that information related to n is missing in the legends of figures 2D; 3B; 4B; EV1A,B,C,D,E; EV2B.

11. Please correct the order and headings of the manuscript sections to: Abstract / The Paper Explained / Introduction /Results / Discussion / Methods / Data Availability / Acknowledgements / Disclosure and Competing Interests Statement / References / Figure Legends / Tables / Expanded View Figure Legends

I look forward to reading a revised version of your manuscript as soon as possible.

Kind regards,
Jingyi

Jingyi Hou
Senior Editor
EMBO Molecular Medicine

*** Instructions to submit your revised manuscript ***

*** PLEASE NOTE *** As part of the EMBO Publications transparent editorial process initiative (see our Editorial at https://www.embopress.org/doi/pdf/10.1002/emmm.201000094), EMBO Molecular Medicine will publish online a Review

Process File to accompany accepted manuscripts.

In the event of acceptance, this file will be published in conjunction with your paper and will include the anonymous referee reports, your point-by-point response and all pertinent correspondence relating to the manuscript. If you do NOT want this file to be published, please inform the editorial office at contact@embomolmed.org.

*****

The authors addressed the remaining editorial issues.

6th Jul 2026

Dear Prof. Jentsch,

We are pleased to inform you that your manuscript is accepted for publication and is now being sent to our publisher to be included in the next available issue of EMBO Molecular Medicine.

Your manuscript will be processed for publication by EMBO Press. It will be copy edited and you will receive page proofs prior to publication. Please note that you will be contacted by Springer Nature Author Services to complete licensing and payment information.

You may qualify for financial assistance for your publication charges - either via a Springer Nature fully open access agreement or an EMBO initiative. Check your eligibility: https://link.springer.com/journal/44321/how-to-publish-with-us

Should you be planning a Press Release on your article, please notify the production team via this form in order to coordinate publication and release dates: https://forms.cloud.microsoft/e/eH4A8JVP6g

If you have any questions, please do not hesitate to contact the Editorial Office. Thank you for your contribution to EMBO Molecular Medicine.

Kind regards,
Jingyi

Jingyi Hou
Senior Editor
EMBO Molecular Medicine

>>> Please note that it is EMBO Molecular Medicine policy for the transcript of the editorial process (containing referee reports and your response letter) to be published as an online supplement to each paper. If you do NOT want this, you will need to inform the Editorial Office via email immediately. More information is available here: https://link.springer.com/partners/embo-press/editorial-policies#Peer%20review

EMBO Press Author Checklist

Corresponding Author Name: Thomas J Jentsch	
Journal Submitted to: EMBO Molecular Medicine	
Manuscript Number: EMM-2026-23864	

USEFUL LINKS FOR COMPLETING THIS FORM
The EMBO Journal - Author Guidelines
EMBO Reports - Author Guidelines
Molecular Systems Biology - Author Guidelines
EMBO Molecular Medicine - Author Guidelines

Reporting Checklist for Life Science Articles (updated January
This checklist is adapted from Materials Design Analysis Reporting (MDAR) Checklist for Authors. MDAR establishes a minimum set of requirements in transparent reporting in the life sciences (see Statement of Task: 10.31222/osf.io/9sm4x). Please follow the journal's guidelines in preparing your
Please note that a copy of this checklist will be published alongside your article.

Abridged guidelines for figures
1. Data
The data shown in figures should satisfy the following conditions:
- the data were obtained and processed according to the field's best practice and are presented to reflect the results of the experiments in an accurate and unbiased manner.
- ideally, figure panels should include only measurements that are directly comparable to each other and obtained with the same assay.
- plots include clearly labeled error bars for independent experiments and sample sizes. Unless justified, error bars should not be shown for technical
- if n<5, the individual data points from each experiment should be plotted. Any statistical test employed should be justified.
- Source Data should be included to report the data underlying figures according to the guidelines set out in the authorship guidelines on Data

2. Captions
Each figure caption should contain the following information, for each panel where they are relevant:
- a specification of the experimental system investigated (eg cell line, species name).
- the assay(s) and method(s) used to carry out the reported observations and measurements.
- an explicit mention of the biological and chemical entity(ies) that are being measured.
- an explicit mention of the biological and chemical entity(ies) that are altered/varied/perturbed in a controlled manner.
- the exact sample size (n) for each experimental group/condition, given as a number, not a range;
- a description of the sample collection allowing the reader to understand whether the samples represent technical or biological replicates (including how many animals, litters, cultures, etc.).
- a statement of how many times the experiment shown was independently replicated in the laboratory.
- definitions of statistical methods and measures:
  - common tests, such as t-test (please specify whether paired vs. unpaired), simple χ2 tests, Wilcoxon and Mann-Whitney tests, can be unambiguously identified by name only, but more complex techniques should be described in the methods section;
  - are tests one-sided or two-sided?
  - are there adjustments for multiple comparisons?
  - exact statistical test results, e.g., P values = x but not P values < x;
  - definition of 'center values' as median or average;
  - definition of error bars as s.d. or s.e.m.

Please complete ALL of the questions below.
Select "Not Applicable" only when the requested information is not relevant for your study.

Materials

Newly Created Materials	Information included in the manuscript?	In which section is the information available? (Reagents and Tools Table, Materials and Methods, Figures, Data Availability Section)
New materials and reagents need to be available; do any restrictions apply?	Yes	Materials and Methods, Data Availability

Antibodies	Information included in the manuscript?	In which section is the information available? (Reagents and Tools Table, Materials and Methods, Figures, Data Availability Section)
For **antibodies** provide the following information:<br>- Commercial antibodies: RRID (if possible) or supplier name, catalogue number and or/clone number<br>- Non-commercial: RRID or citation	Yes	Reagents and Tools table

DNA and RNA sequences	Information included in the manuscript?	In which section is the information available? (Reagents and Tools Table, Materials and Methods, Figures, Data Availability Section)
**Short novel DNA or RNA including primers, probes**: provide the sequences.	Yes	Mutagenesis primers are listed in Appendix Table S3

Cell materials	Information included in the manuscript?	In which section is the information available? (Reagents and Tools Table, Materials and Methods, Figures, Data Availability Section)
**Cell lines:** Provide species information, strain. Provide accession number in repository **OR** supplier name, catalog number, clone number, and/**OR** RRID.	Yes	Reagents and Tools table
**Primary cultures:** Provide species, strain, sex of origin, genetic modification status.	Not Applicable	
Report if the cell lines were recently **authenticated** (e.g., by STR profiling) and tested for mycoplasma contamination.	Yes	Reagents and Tools table

Experimental animals	Information included in the manuscript?	In which section is the information available? (Reagents and Tools Table, Materials and Methods, Figures, Data Availability Section)
**Laboratory animals or Model organisms:** Provide species, strain, sex, age, genetic modification status. Provide accession number in repository **OR** supplier name, catalog number, clone number, **OR** RRID.	Not Applicable	
**Animal observed in or captured from the field:** Provide species, sex, and age where possible.	Not Applicable	
Please detail **housing and husbandry conditions**.	Not Applicable	

Plants and microbes	Information included in the manuscript?	In which section is the information available? (Reagents and Tools Table, Materials and Methods, Figures, Data Availability Section)
**Plants:** provide species and strain, ecotype and cultivar where relevant, unique accession number if available, and source (including location for collected wild specimens).	Not Applicable	
**Microbes:** provide species and strain, unique accession number if available, and source.	Not Applicable	

Human research participants	Information included in the manuscript?	In which section is the information available? (Reagents and Tools Table, Materials and Methods, Figures, Data Availability Section)
If collected and within the bounds of privacy constraints report on age, sex and gender or ethnicity for all study participants.	Yes	Table 1, Appendix Tables S1 and S2

Core facilities	Information included in the manuscript?	In which section is the information available? (Reagents and Tools Table, Materials and Methods, Figures, Data Availability Section)
If your work benefited from core facilities, was their service mentioned in the acknowledgments section?	Not Applicable	

Design

Study protocol	Information included in the manuscript?	In which section is the information available? (Reagents and Tools Table, Materials and Methods, Figures, Data Availability Section)
If study protocol has been **pre-registered, provide DOI in the manuscript**. For clinical trials, provide the trial registration number **OR** cite DOI.	Not Applicable	
Report the **clinical trial registration number** (at ClinicalTrials.gov or equivalent), where applicable.	Not Applicable	

Laboratory protocol	Information included in the manuscript?	In which section is the information available? (Reagents and Tools Table, Materials and Methods, Figures, Data Availability Section)
Provide DOI OR other citation details if **external detailed step-by-step protocols** are available.	Not Applicable	

Experimental study design and statistics	Information included in the manuscript?	In which section is the information available? (Reagents and Tools Table, Materials and Methods, Figures, Data Availability Section)
Include a statement about **sample size** estimate even if no statistical methods were used.	Yes	Figures, Materials and Methods
Were any steps taken to minimize the effects of subjective bias when allocating animals/samples to treatment (e.g. **randomization procedure**)? If yes, have they been described?	Yes	Figures
Include a statement about **blinding** even if no blinding was done.	Yes	Materials and Methods
Describe **inclusion/exclusion criteria** if samples or animals were excluded from the analysis. Were the criteria pre-established?<br><br>If sample or data points were omitted from analysis, report if this was due to attrition or intentional exclusion and provide justification.	Yes	Materials and Methods
For every figure, are **statistical tests** justified as appropriate? Do the data meet the assumptions of the tests (e.g., normal distribution)? Describe any methods used to assess it. Is there an estimate of variation within each group of data? Is the variance similar between the groups that are being statistically compared?	Yes	Figure legends

Sample definition and in-laboratory replication	Information included in the manuscript?	In which section is the information available? (Reagents and Tools Table, Materials and Methods, Figures, Data Availability Section)
In the figure legends: state number of times the experiment was **replicated** in laboratory.	Yes	Figures, Materials and Methods
In the figure legends: define whether data describe **technical or biological replicates**.	Yes	Figures, Materials and Methods

Ethics

Ethics	Information included in the manuscript?	In which section is the information available? (Reagents and Tools Table, Materials and Methods, Figures, Data Availability Section)
Studies involving **human participants**: State details of **authority granting ethics approval** (IRB or equivalent committee(s), provide reference number for approval.	Yes	Materials and Methods
Studies involving **human participants**: Include a statement confirming that **informed consent** was obtained from all subjects and that the experiments conformed to the principles set out in the WMA Declaration of Helsinki and the Department of Health and Human Services Belmont Report.	Yes	Materials and Methods
Studies involving **human participants: For** publication of **patient photos**, include a statement confirming that consent to publish was obtained.	Not Applicable	
Studies involving experimental **animals**: State details of **authority granting ethics approval** (IRB or equivalent committee(s), provide reference number for approval. Include a statement of compliance with ethical regulations.	Not Applicable	
Studies involving **specimen and field samples:** State if relevant **permits** obtained, provide details of authority approving study; if none were required, explain why.	Not Applicable	

Dual Use Research of Concern (DURC)	Information included in the manuscript?	In which section is the information available? (Reagents and Tools Table, Materials and Methods, Figures, Data Availability Section)
Could your study fall under dual use research restrictions? Please check biosecurity documents and list of **select agents and toxins** (CDC): https://www.selectagents.gov/sat/list.htm	Not Applicable	
If you used a select agent, is the security level of the lab appropriate and reported in the manuscript?	Not Applicable	
If a study is subject to dual use research of concern regulations, is the name of the **authority granting approval and reference number** for the regulatory approval provided in the manuscript?	Not Applicable	

Reporting

The MDAR framework recommends adoption of discipline-specific guidelines, established and endorsed through community initiatives. Journals have their own policy about requiring specific guidelines and recommendations to complement MDAR.

Adherence to community standards	Information included in the manuscript?	In which section is the information available? (Reagents and Tools Table, Materials and Methods, Figures, Data Availability Section)
State if relevant guidelines or checklists (e.g., **ICMJE, MIBBI, ARRIVE, PRISMA**) have been followed or provided.	Not Applicable	
For **tumor marker prognostic studies,** we recommend that you follow the **REMARK** reporting guidelines (see link list at top right). See author guidelines, under 'Reporting Guidelines'. Please confirm you have followed these guidelines.	Not Applicable	
For **phase II and III randomized controlled trials**, please refer to the **CONSORT** flow diagram (see link list at top right) and submit the CONSORT checklist (see link list at top right) with your submission. See author guidelines, under 'Reporting Guidelines'. Please confirm you have submitted this list.	Not Applicable	

Data Availability

Data availability	Information included in the manuscript?	In which section is the information available? (Reagents and Tools Table, Materials and Methods, Figures, Data Availability Section)
Have **primary datasets** been deposited according to the journal's guidelines (see 'Data Deposition' section) and the respective accession numbers provided in the Data Availability Section?	Not Applicable	
Were **human clinical and genomic datasets** deposited in a public access-controlled repository in accordance to ethical obligations to the patients and to the applicable consent agreement?	Not Applicable	
Are **computational models** that are central and integral to a study available without restrictions in a machine-readable form? Were the relevant accession numbers or links provided?	Not Applicable	
If publicly available data were reused, provide the respective **data citations in the reference list**.	Not Applicable	
