# Supplementary material for: Abnormal ClC-3/TMEM9-mediated endosomal ion transport in CLCN3-associated neurodevelopmental disease
Source: EMBO Mol Med. 2026 Jul 22;18(9):3474–94. doi: 10.1038/s44321-026-00486-6 (PMC13562645; doi:10.1038/s44321-026-00486-6)
Supplement: Supplementary file 2 [file 44321_2026_486_MOESM8_ESM.pdf]

Expanded View Figures

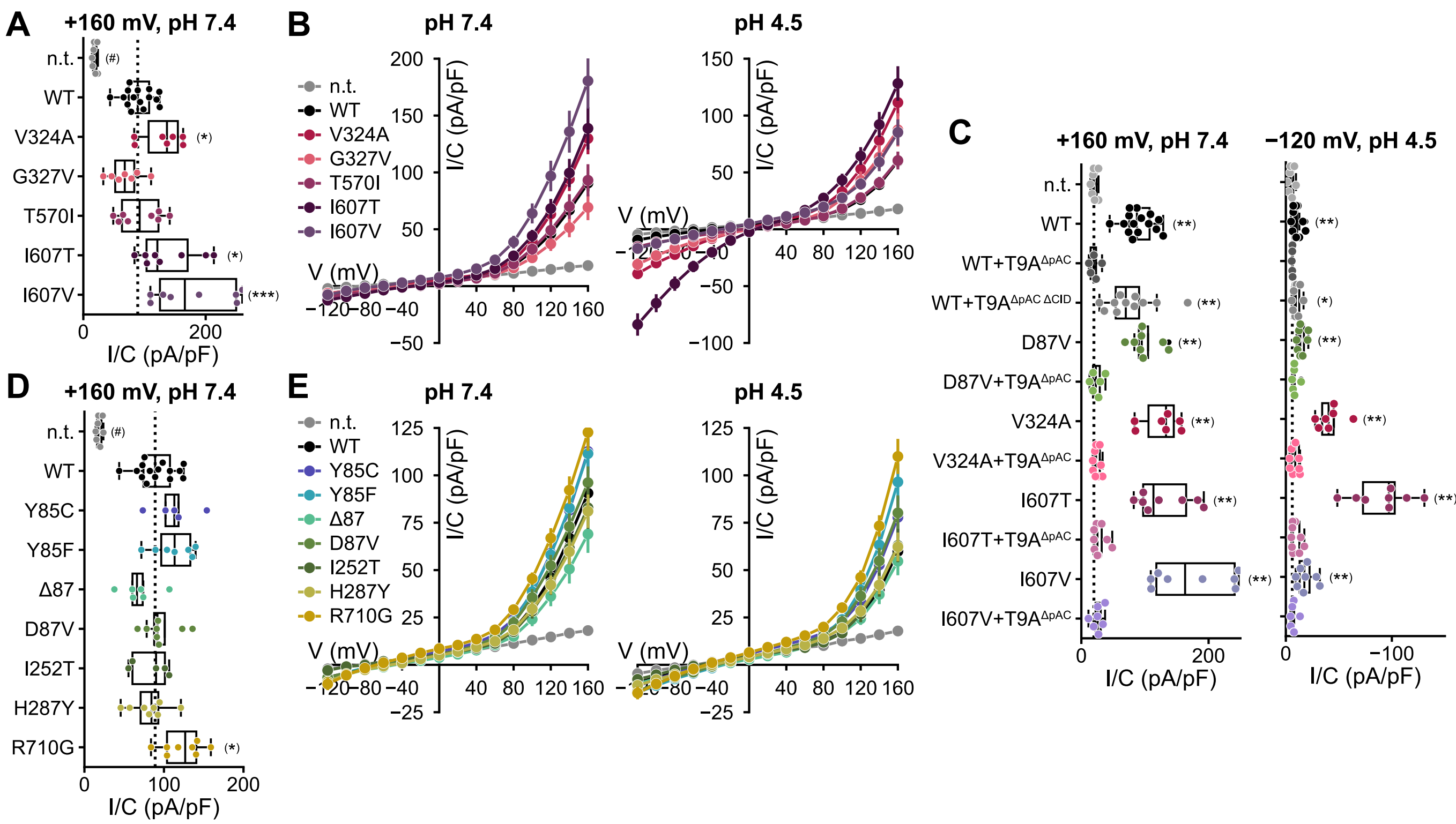

Figure EV1. Detailed current parameters of variants found to be T9A-resistant in the vacuolization assay.

Additional analysis of variants with large inward currents at acidic pH, shown in Fig. 2C,D, and variants with smaller plasma-membrane current changes but T9A-resistant vacuolization, shown in Fig. 3A, B. (**A**) Current densities at +160 mV, bath pH 7.4, for variants with large inward currents obtained from the same recordings as in Fig. 2C,D. (**B**) Current-voltage relationships for the variants shown in (**A**) at bath pH 7.4 (left) and pH 4.5 (right) from the same recordings as in Fig. 2C,D. (**C**) Current densities of WT or mutant ClC-3 expressed alone or with $T9A^{\Delta pAC}$ or $T9A^{\Delta pAC,\Delta CID}$, measured at +160 mV, pH 7.4 (left) and −120 mV, pH 4.5 (right) obtained from the same recordings as in Figs. 2C,E and 3A. (**D**) Current densities at +160 mV, bath pH 7.4, for variants with small plasma-membrane current changes obtained from the same recordings as in Fig. 3A,B. (**E**) Current–voltage relationships for the variants shown in (**D**) at bath pH 7.4 (left) and pH 4.5 (right). Boxes indicate medians and quartiles; dotted lines indicate the median WT current density in (**A**) and (**D**), or in (**C**), the median WT + $T9A^{\Delta pAC}$ current density. In (**B**, **E**), error bars, SEM. In (**A**, **D**), WT and n.t. data are reproduced from Fig 2D for comparison; $^{*}P < 0.05$; $^{***}P < 0.001$; $^{\#}P < 0.0001$ against WT; in (**C**), $^{**}P < 0.01$ against WT + $T9A^{\Delta pAC}$ (Mann–Whitney *U* test; false discovery rate was controlled using the Benjamini–Hochberg procedure).

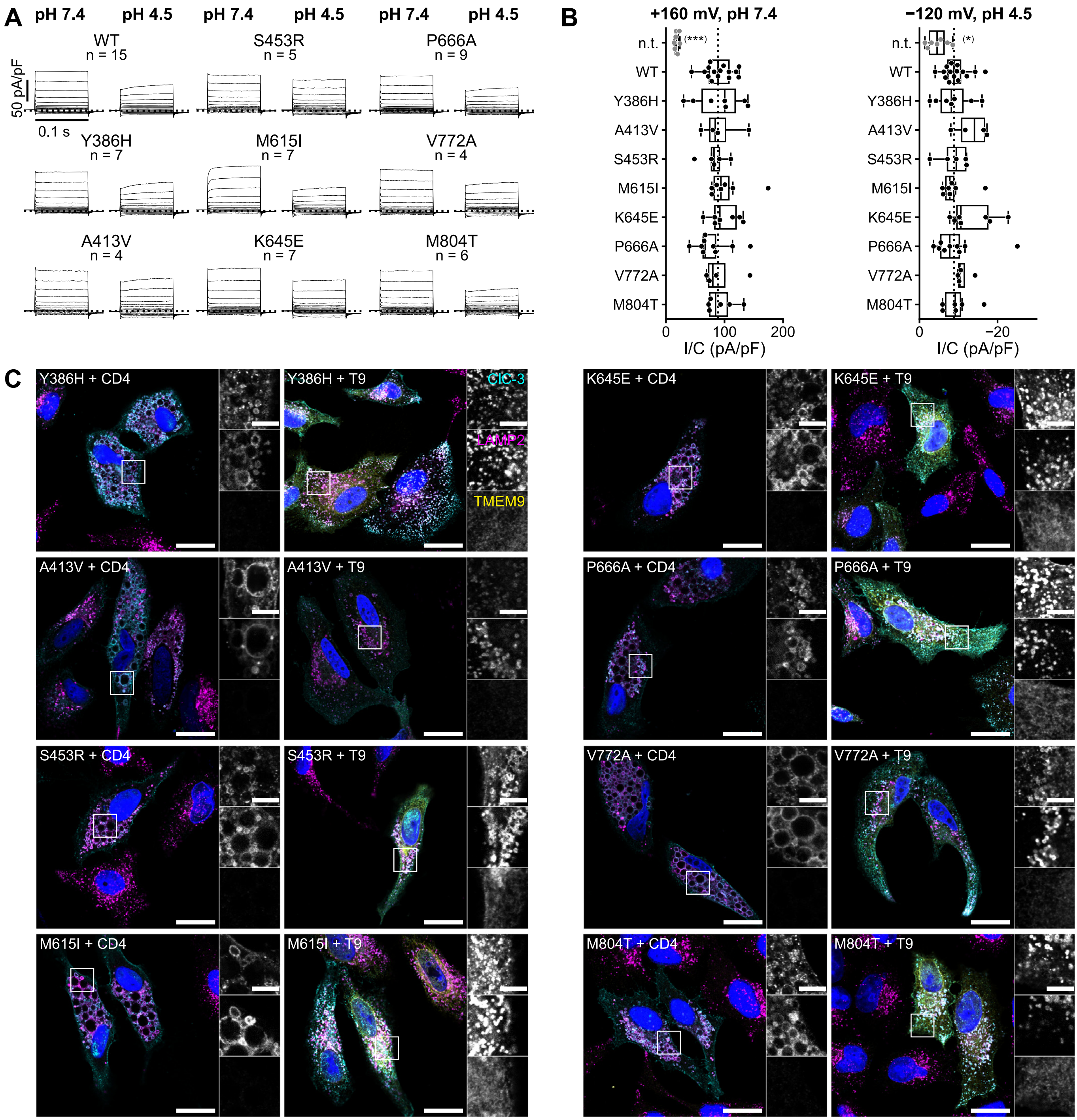

Figure EV2. Variants lacking functional effects in our assays.

p.Tyr386His, p.Ala413Val, p.Ser453Arg, p.Met615Ile, p.Met604Thr, p.Lys645Glu, p.Pro666Ala, p.Val772Ala, and p.Met804Thr retain sensitivity to T9A-mediated inhibition and do not elicit significant inward currents at acidic bath pH. (**A**) Averaged whole-cell current traces of ClC-3 variants obtained at bath pH 7.4 (left) and 4.5 (right) as in Fig. 2A, WT data are reproduced from the same panel for comparison. Dotted lines indicate zero current density. (**B**) Current densities at +160 mV, bath pH 7.4 (left) and −120 mV, bath pH 4.5 (right). Boxes indicate the median and quartiles of the distribution. The dotted line indicates the median WT current density. WT and n.t. data are reproduced from Fig. 2C for comparison. Recordings were obtained from n cells per variant, with *n* indicated in (**A**), across at least three independent transfections. *$P < 0.05$; ***$P < 0.001$ (against WT, Mann–Whitney *U* test; false discovery rate was controlled using the Benjamini–Hochberg procedure). (**C**) Immunofluorescent staining of cells overexpressing ClC-3 variants with T9A (right) or CD4 (left). Scale bars on the merged panels, 20 μm; scale bars on the close-ups, 5 μm. *CLCN3* variants p.Ala413Val, p.Ser453Arg, and p.Val772Ala and associated clinical symptoms have been described before (Duncan et al, 2021), whereas the other variants (p.Tyr386His, p.Met615Ile, p.Lys645Glu, p.Pro666Ala, p.Met804Thr) are newly described here.

 © The Author(s)

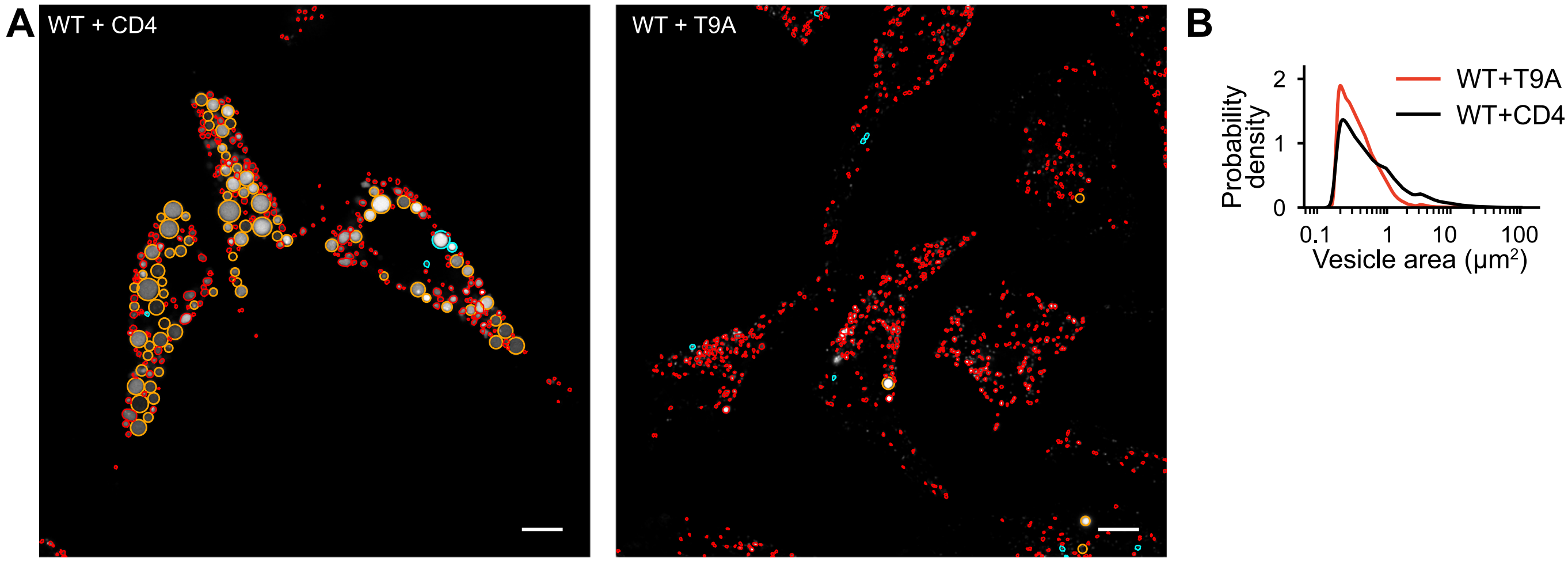

Figure EV3. Quantification of cell vacuolization upon ClC-3 overexpression.

(**A**) WT HeLa cells overexpressing WT ClC-3 with CD4 control or T9A stained with Lysosensor® Yellow/Blue and imaged at pH-insensitive wavelength. Large vesicles, for which a circular fit was performed, are outlined in orange, whereas the rest of the vesicles detected by the algorithm are shown in red. Detections with top 1% relative standard deviation of intensity are highlighted in cyan and were excluded from analysis. (**B**) Estimated area of Lysosensor-positive vesicles in HeLa cells overexpressing WT ClC-3 with T9A (red) or CD4 (black) as evaluated by our script (see "Methods"). Size distribution was obtained by pooling all lysosomes from >5 fields of view from 13 independent transfections.

© The Author(s)
